# A phosphorylation switch in the Mediator MED15 controls cellular senescence and cognitive decline

**DOI:** 10.1038/s41421-025-00820-1

**Published:** 2025-08-19

**Authors:** Haozheng Li, Yuanming Zheng, Chunlei Yuan, Jiayi Wang, Xiaying Zhao, Ming Yang, Defei Xiong, Yenan Yang, Yunpeng Dai, Yiming Gao, Yuqi Wang, Lei Xue, Gang Wang

**Affiliations:** 1 State Key Laboratory of Genetics and Development of Complex Phenotypes, School of Life Sciences and Zhongshan Hospital, Fudan University, Shanghai, China; 2 School of Biological Sciences, The University of Hong Kong, Hong Kong, China; 3 School of Life Sciences, Westlake University, Hangzhou, Zhejiang China

**Keywords:** Senescence, Phosphorylation

## Abstract

A hallmark of aging is chronic systemic inflammation, which is exacerbated by the hypersecretory aging phenotype known as the senescence-associated secretory phenotype (SASP). How the SASP is initiated to accelerate tissue inflammation and aging is an outstanding question in aging biology. Here, we showed that phosphorylation of the Mediator subunit MED15 at T603 is able to control the SASP and aging. Transforming growth factor-β selectively induces CDK1-mediated MED15 T603 phosphorylation to control SASP gene expression. The MED15 T603 dephosphorylated mutant (T603A) inhibits the SASP and cell senescence, whereas the T603 phosphorylation-mimicking mutant (T603D) has the opposite effect. Mechanistically, forkhead box protein A1 preferentially binds to unphosphorylated but not phosphorylated MED15 at T603 to suppress SASP gene expression. Notably, aging mice harboring dephosphorylated mutation in this phosphosite exhibit improved learning and memory through the attenuation of the SASP across tissues. Overall, our study indicates that MED15 T603 phosphorylation serves as a control switch for SASP production, which underlies tissue aging and cognitive decline and provides a novel target for age-related pathogenesis.

## Introduction

Aging is broadly defined as functional decline that occurs throughout the whole body. The accumulation of senescent cells is considered a hallmark of aging and is thought to contribute to various aging pathologies^1–3^. Cellular senescence is a state of cell cycle arrest accompanied by the secretion of chemokines, cytokines, matrix-remodeling proteases and other molecules^4,5^. This hypersecretory phenotype is known as the senescence-associated secretory phenotype (SASP)^6^. The SASP is a highly heterogeneous program, and its composition is influenced by a range of intrinsic and extrinsic factors^7,8^. The SASP has been hypothesized to link cellular senescence and inflammation^9^ and to participate in tissue dysfunction. The accumulation of senescent cells with the SASP is associated with increased susceptibility to several age-related diseases^10^, including neurodegenerative diseases, cardiovascular diseases, metabolic disorders, and immune system diseases^11^. In normal cells, SASP genes are usually highly inhibited to prevent the inappropriate activation of inflammatory signals, whereas in senescent cells, the SASP genes are highly expressed^12^. How SASP gene expression is activated to trigger cellular senescence, inflammation and the aging process remains to be actively studied.

The Mediator complex is a master transcriptional cofactor comprising ~30 subunits in mammalian cells^13^ and links extracellular signals to the basal transcriptional machinery for selective target gene expression^14^. In response to various environmental or developmental cues, distinct transcription factors interact with different Mediator subunits to control diverse biological processes^15^. MED1, for example, interacts with PPARγ2 to regulate adipogenesis^16^, and MED12 interacts with β-catenin to relay the Wnt signaling^17^ and gastrulation^18^. The interaction between MED16 and NRF2 plays a crucial role in ROS production and redox homeostasis^19,20^. We previously reported that MED23 controls lipid metabolism and obesity^21^ and participates in the RoRα-regulated liver inflammatory response and liver fibrosis^22^. A single-site mutation of MED23 (R617Q) leads to intellectual disability by altering the selective chromatin conformation and enhancer activities^23^. MED15 modulates transforming growth factor β (TGFβ)/Smad signaling during development^24^ and breast cancer cell metastasis^25^; MED15 deficiency attenuates TGFβ-targeted gene expression and relieves TGFβ-mediated growth inhibition and metastasis^25^. Secreted TGFβ is considered a SASP factor and plays a pivotal role in cellular senescence and age-related diseases, including Alzheimer’s disease, muscle atrophy, fibrosis, and obesity^26–29^. Despite that much has been know about various interactions between transcription factors and Mediator components, less is known about how signaling directly hinges upon the Mediator complex per se; and specifically, how exactly TGFβ signaling impacts on the Mediator to regulate the associated transcription factors and the selective biological processes remains to be further investigated.

In this study, we found that the MED15 T603 phosphosite plays a crucial role in controlling the SASP production and aging. Cellular senescence and the SASP are relieved by the T603A mutation in MED15 but accelerated by the T603D mutation. An integrated analysis revealed that MED15 T603 dephosphorylation delays aging processes through an increased interaction with forkhead box protein A1 (FOXA1) to suppress downstream SASP gene expression. Notably, we generated Med15 T604A mutant mice using a gene editing technique, and these mice exhibit alleviated tissue aging and resistance to cognitive decline, accompanied by decreased serum levels of SASP factors in aging mice compared to wild-type (WT) control mice. Overall, our study revealed a crucial phosphorylation switch in the Mediator complex that controls cellular senescence and tissue aging through the modulation of SASP production.

## Results

### MED15 T603 phosphorylation participates in TGFβ-inhibited cell growth

**Fig. 1:**
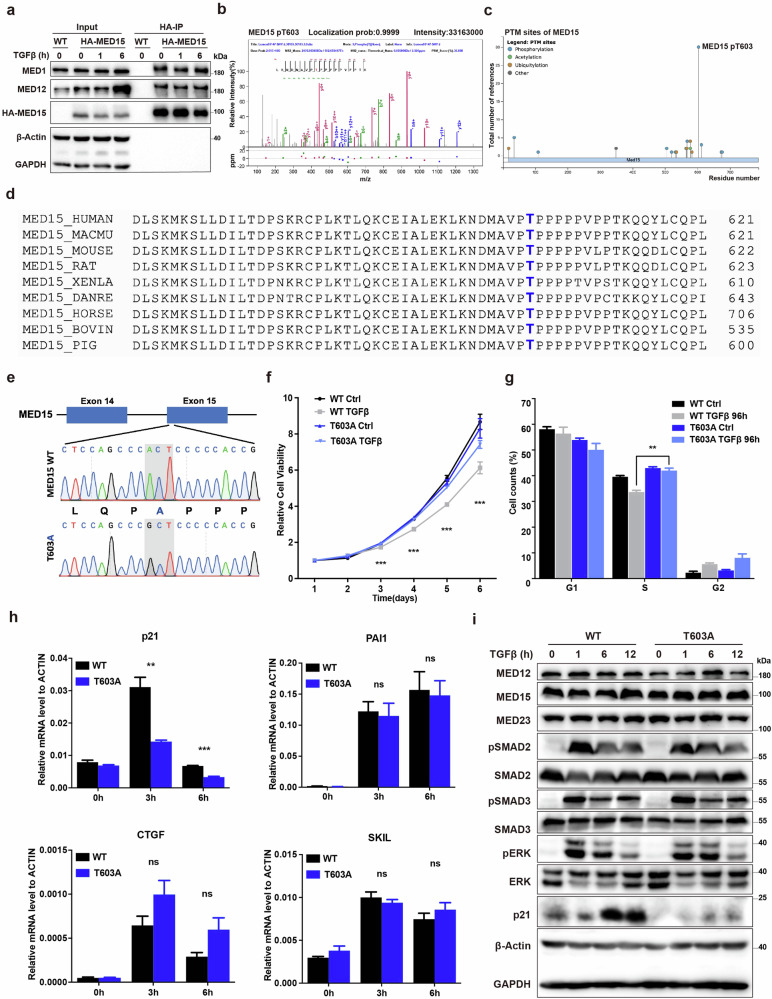
MED15 T603 testified as a phosphorylation site involved in TGFβ-inhibited cell growth. **a** Endogenous co-IP assay using HA-beads to pull down HA-MED15 from 293T cells. The HA tag was knocked into the N-terminus of MED15. **b** Tandem mass spectrometry analysis of the MED15 peptides modified by phosphorylation of the T603 residue. **c** Posttranslational modification analysis of MED15 by PhosphoSitePlus database. **d** Sequence alignment of MED15 proteins across different species. The blue bold T indicates the MED15 T603 site. **e** Endogenous MED15 T603A mutant genotyping via PCR and Sanger sequencing in HaCaT cells. **f** CCK-8 assays were used to assess the proliferation rates of WT and T603A mutant HaCaT cells treated without or with 5 ng/mL TGFβ. The values are presented as means ± SD. ***P < 0.001. **g** Statistical analysis of the cell cycle distribution of WT and T603A mutant HaCaT cells treated without or with 5 ng/mL TGFβ. The values are presented as means ± SD. **P < 0.01. **h** qRT-PCR was used to determine the mRNA levels of the TβR genes in MED15 T603A cells and control cells treated without or with 2 ng/mL TGFβ. The values are presented as means ± SD. **P < 0.01 and ***P < 0.001. **i** Immunoblot analysis with the indicated antibodies in WT and MED15 T603A mutant HaCaT cells treated without or with 2 ng/mL TGFβ. β-Actin and GAPDH were blotted as loading controls.

To understand the mechanism and regulation of TGFβ signaling hinging upon the Mediator complex, we performed immunoprecipitation-mass spectrometry (IP-MS) analysis and identified a novel phosphorylated threonine residue (T603) in MED15 (Fig. 1a, b). The PhosphoSitePlus database also showed that T603 site is a major high-abundance phosphorylation site of MED15 (Fig. 1c). The multiple sequence alignment of amino acids revealed that the T603 site of MED15 is conserved across species (Fig. 1d). We utilized CRISPR-Cas9 gene editing to generate an endogenous MED15 T603A (threonine to alanine) single-site mutant to mimic the MED15 T603 dephosphorylation state in HaCaT cells (Fig. 1e), an immortalized epithelial cell line often used for studying TGFβ/Smad signaling, and to verify the function and relevance of this phosphorylation site to TGFβ signaling. Cell proliferation assays revealed that MED15 T603A mutant cells significantly relieved TGFβ-induced inhibition of cell growth compared to WT cells treated with TGFβ (Fig. 1f). The flow cytometry analysis further revealed that compared to WT cells, the MED15 T603A mutant increased the proportion of cells in the S phase after TGFβ treatment for 96 h (Fig. 1g; Supplementary Fig. S1), suggesting that the MED15 T603A mutation confers resistance to TGFβ-induced G1/S cell cycle arrest.

We next examined whether the MED15 T603A mutant affects TGFβ-responsive gene (TβR gene) expression and TGFβ-induced Smad phosphorylation. The MED15 T603A mutant did not change the response of TβR genes, such as PAI1, CTGF and SKIL, to TGFβ but notably dampened TGFβ-induced p21 expression (Fig. 1h). Western blot analysis revealed that the MED15 T603A mutant consistently inhibited TGFβ-induced p21 expression but did not affect TGFβ-induced Smad2/3 and ERK phosphorylation (Fig. 1i). Additionally, the MED15 T603A mutation did not alter the expression levels of MED15 or other Mediator complex subunits, such as MED12 and MED23 (Fig. 1i). Overall, these findings indicate that the MED15 T603 site is a phosphorylation site that is specifically involved in TGFβ-inhibited cell growth and TGFβ-induced p21 expression.

### The MED15 T603A mutation confers resistance to cellular senescence

**Fig. 2:**
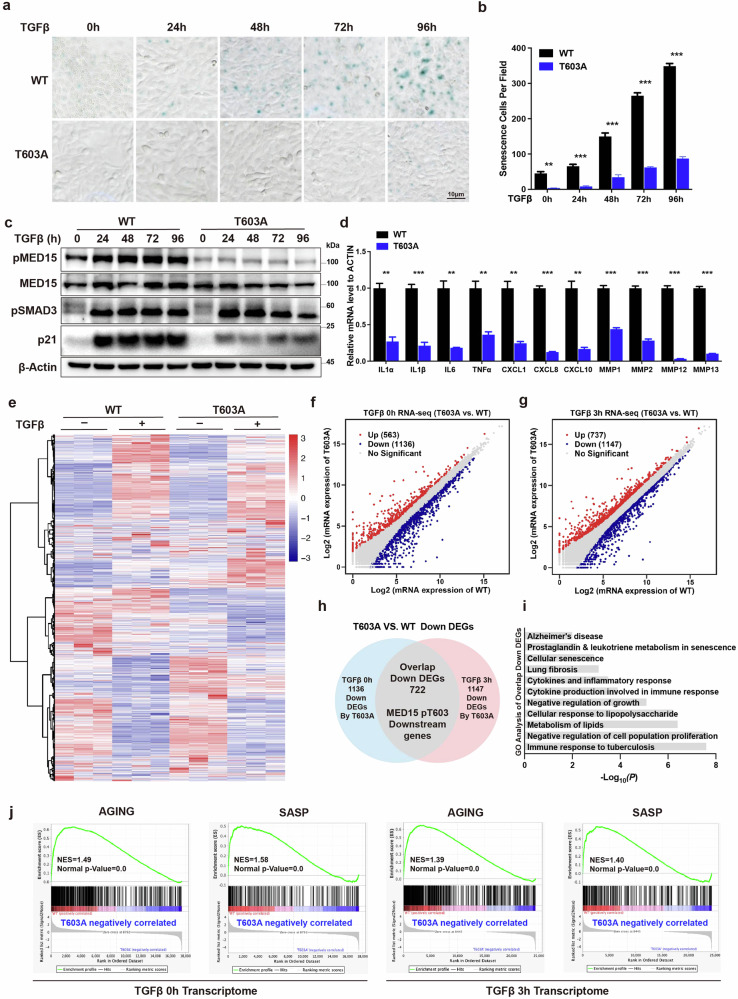
The MED15 T603 dephosphorylated mutation confers resistance to cellular senescence. **a** β-Galactosidase activity was determined by β-gal staining in cultured WT and MED15 T603A mutant HaCaT cells treated without or with 5 ng/mL TGFβ for different periods. **b** Statistical analysis of the senescent cells in (**a**). The values are presented as means ± SD. **P < 0.01 and ***P < 0.001. **c** Immunoblot analysis with the indicated antibodies in WT and MED15 T603A mutant HaCaT cells treated without or with 2 ng/mL TGFβ. β-Actin was blotted as a loading control. The pMED15 antibody specifically targeted the phosphorylated T603 site of MED15. **d** qRT-PCR assays were performed to determine the mRNA levels of SASP genes in WT and MED15 T603A mutant HaCaT cells. The values are presented as means ± SD. **P < 0.01 and ***P < 0.001. **e** Heatmap of the RNA-seq analysis of DEGs in WT and MED15 T603A mutant HaCaT cells treated without or with 2 ng/mL TGFβ for 3 h. **f**, **g** Scatter plot of DEGs (fragments per kilobase of transcript per million mapped reads (FPKM), fold change ≥ 1.5, *P* < 0.05) identified by RNA-seq in WT and MED15 T603A mutant HaCaT cells treated without or with 2 ng/mL TGFβ for 3 h. **h** Venn diagram showing the 722 overlapping genes among the downregulated DEGs identified by RNA-seq in the MED15 T603A mutant cells. **i** GO analysis of 722 genes downstream of MED15 pT603. **j** GSEA enrichment plot of the indicated gene sets. NES normalized enrichment score.

As the classical aging marker p21 was downregulated by the MED15 T603A mutant after TGFβ treatment, we hypothesized that the MED15 T603A mutant may affect cell growth and senescence. β-Gal staining was performed to examine senescent cells. As shown in Fig. 2a, b, compared to WT HaCaT cells, MED15 T603A cells exhibited less senescence at different time points following TGFβ treatment. Interestingly, the MED15 T603A mutant also decreased the number of senescent cells even in the absence of TGFβ treatment, suggesting that MED15 T603 dephosphorylation alleviates cell senescence in the steady state.

We next developed a rabbit polyclonal antibody that specifically recognizes the MED15 pT603 site to further investigate the role of MED15 T603 phosphorylation in cellular senescence. TGFβ treatment at different time points increased p21 expression and cellular senescence, accompanied by increased MED15 T603 phosphorylation in WT cells; in contrast, MED15 T603A cells presented minimal changes in the phosphorylation signal with or without TGFβ treatment, underscoring the specificity of the antibody. Moreover, the MED15 T603A mutant did not affect Smad3 phosphorylation in response to TGFβ treatment (Fig. 2c).

Cellular senescence is associated with the SASP, characterized by the upregulation of cytokines, chemokines, growth factors, proteases, and other molecules, which further exacerbates cellular and tissue aging through autocrine and paracrine mechanisms^7^. Compared to WT cells, MED15 T603A cells presented significantly decreased expression of SASP factors, such as IL1α, IL1β, IL6 and TNFα (Fig. 2d). We conducted a genome-wide transcriptome analysis of WT and MED15 T603A mutant HaCaT cells with or without TGFβ treatment (3 h) to better understand the overall impact of the MED15 T603A mutation on cellular senescence and the SASP (Fig. 2e). Principal component analysis revealed distinct clustering of WT and MED15 T603A cells, with closely grouped biological replicates (Supplementary Fig. S2a). In the steady state, 1136 genes were downregulated and 563 genes were upregulated in the MED15 T603A mutant cells compared to WT cells (Fig. 2f; Supplementary Fig. S2b). After 3 h of TGFβ treatment, 1147 genes were downregulated and 737 genes were upregulated in the MED15 T603A mutant HaCaT cells compared to the WT cells (Fig. 2g; Supplementary Fig. S2c). We analyzed how MED15 phosphorylation may regulate the TGFβ signaling pathway by defining a set of 1672 genes as TβR genes, whose expression was upregulated by > 1.5-fold with TGFβ treatment in WT cells. Among the 1672 genes, 1372 TβR genes were similarly upregulated by TGFβ treatment in both WT and T603A cells. However, the expression of 300 TβR genes was specifically attenuated in the MED15 T603A mutant cells in both the steady state and the TGFβ-stimulated state (Supplementary Fig. S2d). Interestingly, various SASP genes, such as IL6, CXCL1 and CXCL8, were among the 300 TβR genes (Supplementary Fig. S2e), suggesting that MED15 T603 dephosphorylation specifically downregulated TGFβ-stimulated SASP gene expression to relieve cellular senescence.

We overlapped the differentially expressed genes (DEGs) downregulated by the MED15 T603A mutation in cells treated with (1147 DEGs) or without (1136 DEGs) TGFβ to better characterize the genes regulated by MED15 T603 phosphorylation and identified 722 genes as target genes of the MED15 T603A mutant, as shown in Fig. 2h. The Gene Ontology (GO) analysis revealed that the 722 downstream genes are associated with the inflammatory response, cell proliferation, cellular senescence and other aging-related pathological processes (Fig. 2i). The gene set enrichment analysis (GSEA) revealed that the MED15 T603A mutant was negatively correlated with aging and the SASP (Fig. 2j). On the other hand, overlapping the genes upregulated by the MED15 T603A mutant in cells treated with (737 DEGs) or without (563 DEGs) TGFβ (Supplementary Fig. S2f) revealed that a set of 341 genes was strongly associated with neurodevelopment, including brain development, neurogenesis, neuron differentiation, cognition and memory (Supplementary Fig. S2g). These data suggest that MED15 T603 dephosphorylation significantly relieves cellular senescence and the SASP gene program, which could be beneficial for neurodevelopment and possibly enhance cognitive ability.

### MED15 T603 phosphorylation accelerates cell senescence by exacerbating the SASP

**Fig. 3:**
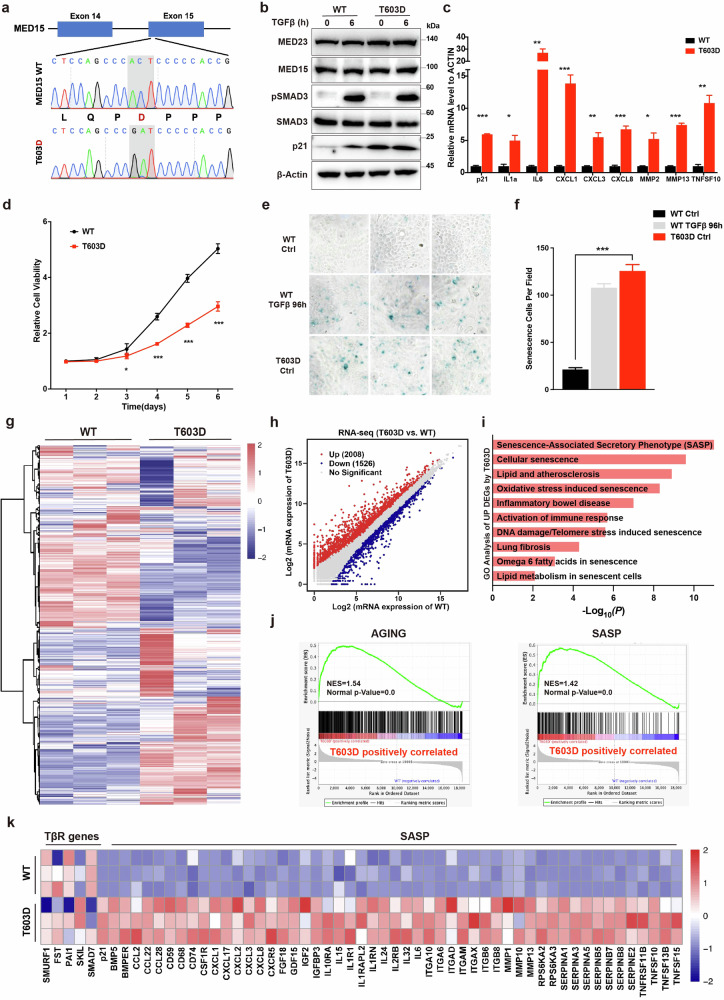
MED15 T603 phosphorylation accelerates cellular senescence by exacerbating the SASP. **a** Endogenous MED15 T603D mutant genotyping by PCR and Sanger sequencing in MCF7 cells. **b** Immunoblot analysis with the indicated antibodies in WT and MED15 T603D mutant MCF7 cells treated without or with 2 ng/mL TGFβ. β-Actin was blotted as a loading control. **c** qRT-PCR was used to determine the mRNA levels of SASP genes in WT and MED15 T603D mutant MCF7 cells. The values are presented as means ± SD. *P < 0.05, **P < 0.01, and ***P < 0.001. **d** CCK-8 assays were performed to assess the proliferation rates of WT and T603D mutant MCF7 cells. The values are presented as means ± SD. *P < 0.05 and ***P < 0.001. **e** β-Galactosidase activity was determined by β-gal staining in cultured WT and MED15 T603D mutant MCF7 cells treated without or with 5 ng/mL TGFβ. **f** Statistical analysis of the senescent cells in **(e)**. The values are presented as means ± SD. ***P < 0.001. **g** Heatmap of DEGs identified by RNA-seq in WT and MED15 T603D mutant MCF7 cells. **h** Scatter plot of DEGs (FPKM, fold change ≥ 1.5, *P* < 0.05) identified by RNA-seq in WT and MED15 T603D mutant MCF7 cells. **i** GO analysis of DEGs upregulated by MED15 T603D mutant. **j** GSEA enrichment plot of the indicated gene sets. **k** Heatmaps of DEGs associated with TβR genes and SASP genes.

To further determine the impact of MED15 T603 phosphorylation on cell senescence, we knocked in the endogenous MED15 T603D (threonine to aspartic acid) single site mutation to mimic the MED15 T603 phosphorylation state in MCF7 cells (Fig. 3a). This mutation could not be acquired in HaCaT cells, as it seemed to cause cell death. Western blot revealed that MED15 T603D did not alter the protein levels of the MED15, MED23 and SMAD3 or TGFβ-induced SMAD3 phosphorylation (Fig. 3b). In contrast to p21 reduction by MED15 T603A, MED15 T603D increased p21 expression, compared to WT, and SASP component such as IL1α, IL6 and CXCL1 was significantly elevated in MED15 T603D cells as well (Fig. 3c). Further analysis revealed decreased growth of the MED15 T603D cells (Fig. 3d) and a reduced number of S-phase cells compared to WT cells (Supplementary Fig. S3a, b). In contrast to the finding that MED15 T603A conferred resistance to cell senescence, MED15 T603D in MCF7 cells dramatically accelerated spontaneous senescence in the absence of TGFβ treatment, as indicated by β-gal staining (Fig. 3e, f), further supporting the strong connection between MED15 T603 phosphorylation and cellular senescence.

An RNA-seq analysis was subsequently performed and revealed substantial changes between the transcriptomes of the MED15 T603D mutant and WT cells (Fig. 3g; Supplementary Fig. S3c). A total of 3534 genes were differentially expressed by at least 1.5-fold, with 2008 genes upregulated and 1526 genes downregulated by the MED15 T603D mutation in MCF7 cells (Fig. 3h; Supplementary Fig. S3d). Contrary to the MED15 T603A mutant, the GO analysis revealed that the genes whose expression was upregulated in MED15 T603D cells were associated with the SASP and cellular senescence (Fig. 3i), whereas the genes whose expression was downregulated were related to neurodevelopment (Supplementary Fig. S3e). GSEA also revealed that the MED15 T603D mutation was positively correlated with aging and the SASP (Fig. 3j). Consistently, several age-related diseases, such as lung cancer, atherosclerosis, and Alzheimer’s disease, were predicted to be associated with upregulated genes in MED15 T603D mutant cells (Supplementary Fig. S3f).

Interestingly, similar to the T603A mutant, the expression of canonical TβR genes such as SMURF1, FST, PAI1, SKIL and SMAD7 was largely unchanged by the MED15 T603D mutant. However, in contrast to T603A, the expression of p21 and many SASP genes was significantly increased in the MED15 T603D mutant, based on the RNA-seq data (Fig. 3k). Collectively, these findings underscore a positive relationship between MED15 T603 phosphorylation and cellular senescence, as evidenced by the finding that T603 phosphorylation-mimicking mutant accelerates cellular senescence through the upregulation of the SASP.

### CDK1 is the kinase that catalyzes MED15 T603 phosphorylation

**Fig. 4:**
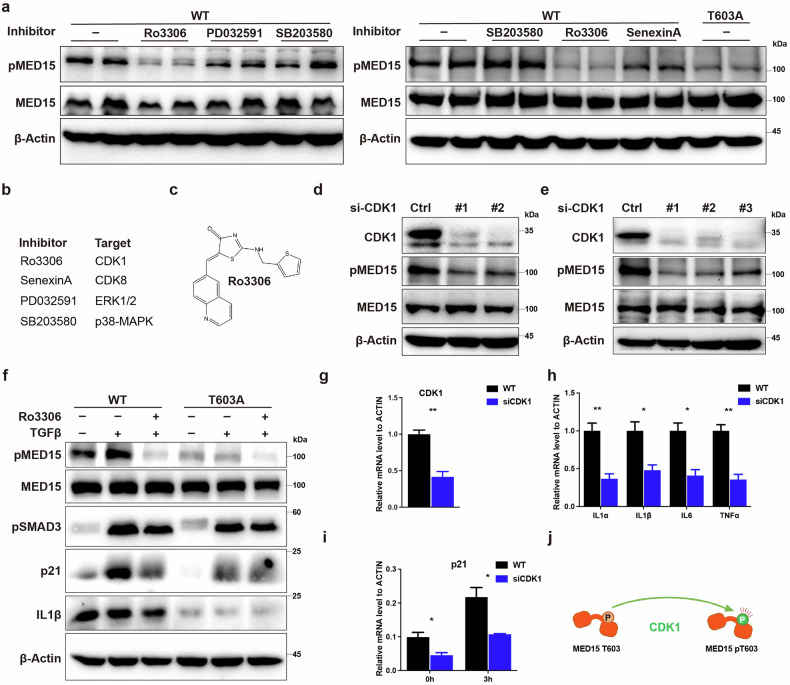
CDK1 is the upstream kinase of MED15 T603 phosphorylation. **a** Immunoblot analysis using the indicated antibodies in HaCaT cells treated without or with kinase inhibitors. **b** Kinase inhibitors and their target kinases. **c** The chemical structure of Ro3306. **d** Immunoblot analysis with the indicated antibodies in HaCaT cells transfected without or with si-CDK1. **e** Immunoblot analysis with the indicated antibodies in HeLa cells transfected without or with si-CDK1. **f** Immunoblot analysis with the indicated antibodies in WT and T603A mutant HaCaT cells treated without or with 10 μM Ro3306 and 2 ng/mL TGFβ. **g** qRT-PCR assays were performed to determine the mRNA levels of CDK1 in HaCaT cells transfected without or with si-CDK1. The values are presented as means ± SD. **P < 0.01. **h** qRT-PCR assays were used to determine the mRNA levels of SASP genes in HaCaT cells transfected without or with si-CDK1. The values are presented as means ± SD. *P < 0.05 and **P < 0.01. **i** qRT-PCR assays were used to determine the mRNA levels of p21 in HaCaT cells treated without or with si-CDK1 and TGFβ. The values are presented as means ± SD. *P < 0.05. **j** Schematic model showing that CDK1 phosphorylates MED15 at T603.

TGFβ-induced cell senescence is directly linked to MED15 T603 phosphorylation, which is inhibited by the MED15 T603A mutation. Various kinase inhibitors, including SenexinA (CDK8), PD032591 (ERK1/2) and SB203580 (p38-MAPK), were screened to determine the kinase involved in T603 phosphorylation. None of these inhibitors, except Ro3306 (CDK1), effectively inhibited MED15 T603 phosphorylation (Fig. 4a–c), suggesting that CDK1 could be the upstream kinase responsible for MED15 T603 phosphorylation. RNA interference (RNAi)-mediated knockdown of CDK1 was performed in both HeLa (Fig. 4d) and HaCaT cells (Fig. 4e) to exclude a nonspecific inhibitory effect of Ro3306, and MED15 T603 phosphorylation was significantly decreased in both cell lines without effects on total MED15 protein levels. Since TGFβ stimulates MED15 T603 phosphorylation, we further investigated whether CDK1 inhibition could suppress TGFβ-induced MED15 T603 phosphorylation. TGFβ treatment of HaCaT cells incubated with or without Ro3306 revealed that MED15 T603 phosphorylation increased 3 h after TGFβ treatment but was significantly reduced by Ro3306 addition. As expected, the T603A mutant presented minimal basal phosphorylation of MED15, and Ro3306 treatment further abolished the basal phosphorylation of MED15. The levels of p21 and the SASP factor IL1β were also decreased by CDK1 inhibition and abolished by MED15 T603A mutation in HaCaT cells (Fig. 4f). Moreover, CDK1 knockdown similarly suppressed p21 and SASP factor expression (Fig. 4g–i). Overall, we found that CDK1 is likely the upstream kinase involved in MED15 T603 phosphorylation (Fig. 4j).

### FOXA1 interacts with MED15 dephosphorylated at T603 to suppress SASP gene expression

**Fig. 5:**
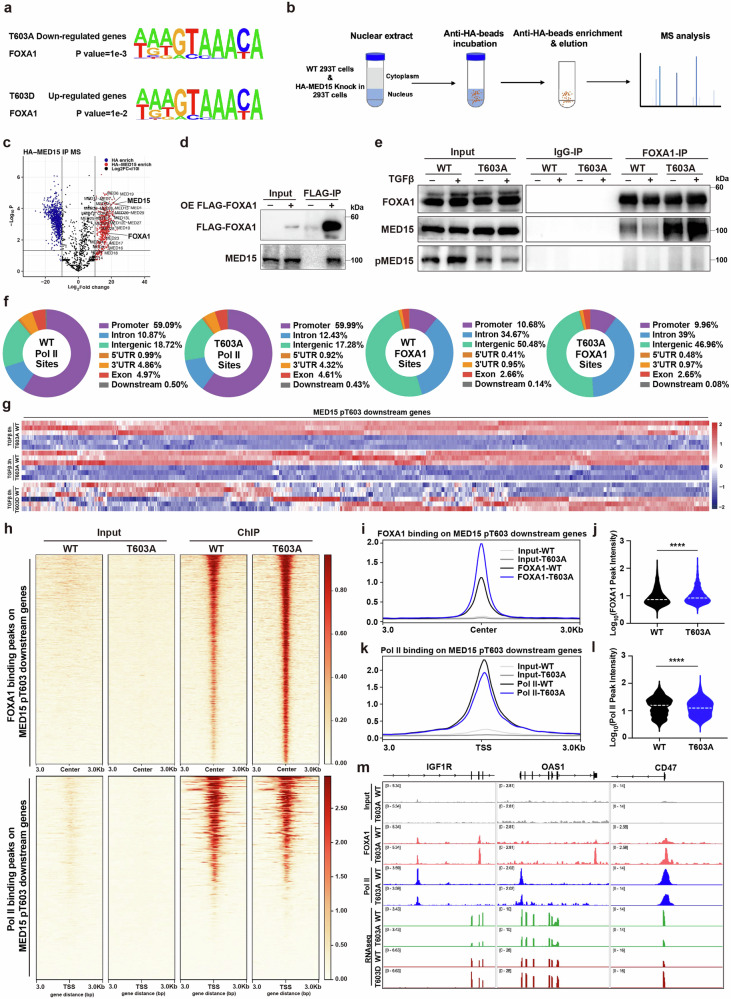
MED15 T603 phosphorylation regulates senescence by modulating FOXA1-mediated repression of downstream genes. **a** Transcription factor motif analysis of T603A-downregulated genes and T603D-upregulated genes identified via RNA-seq**. b** Workflow of HA IP-MS in HA-MED15 knock-in and WT cell nuclear extracts**. c** Scatter plot of three HA IP-MS replicates in terms of log_2_ (fold change) correlation and log_10_ (*P* value) for three sets of experiments: HA IP of HA-MED15 293T cells vs 293T WT control cells. IP experiments were performed with nuclear extracts isolated from 293T cells (*n* = 3)**. d** Co-IP assay using FLAG beads to pull down FLAG-FOXA1 in FLAG-FOXA1-overexpressing and control 293T cells**. e** Endogenous co-IP assay using a FOXA1 antibody to pull down MED15 and pMED15 in WT and T603A mutant HaCaT cells treated without or with TGFβ for 3 h**. f** Pie chart showing Pol II and FOXA1 ChIP-seq binding sites, promoter regions defined as −1000 bp to +200 bp relative to the transcription start site (TSS). *n* = 2 biologically independent ChIP-seq replicates in WT and T603A mutant HaCaT cells**. g** Heatmap of the expression of MED15 pT603 downstream genes in MED15 T603A mutant HaCaT cells and MED15 T603D mutant MCF7 cells**. h** Heatmap of Pol II and FOXA1 binding peaks on MED15 pT603 downstream genes identified via ChIP-seq. **i** Average enrichment profiles of FOXA1 ChIP-seq signals at MED15 pT603 downstream genes in the reference genome hg19. ChIP-seq signals are normalized to CPM (counts per million mapped reads)**. j** Violin plot of the FOXA1 binding intensity to MED15 pT603 downstream genes. ****P < 0.0001, *t*-test. **k** Average enrichment profiles of Pol II ChIP-seq signals at MED15 pT603 downstream genes in the reference genome hg19. ChIP-seq signals are normalized to CPM. **l** Violin plot of the Pol II binding intensity to MED15 pT603 downstream genes. ****P < 0.0001, Kolmogorov–Smirnov (K–S) test. **m** ChIP-seq track plot showing FOXA1 and Pol II genomic binding associated with the IGF1R, OAS1 and CD47 genes in WT and T603A HaCaT cells. The RNA-seq track plot shows corresponding changes in gene expression in cells expressing different MED15 T603 mutants. ChIP-seq and RNA-seq signals are normalized to CPM.

To investigate the molecular mechanism through which MED15 T603 phosphorylation regulates senescence, we hypothesized that different MED15 T603 phosphorylation states may distinctly impact on key transcription factors to control the cellular senescence. As shown in Fig. 5a, we performed a promoter motif analysis of genes downregulated by the MED15 T603A mutant (1136 genes) and upregulated by the MED15 T603D mutant (2008 genes) to identify potentially relevant transcription factors. FOXA1 was significantly enriched in both sets of genes. We subsequently performed IP-MS (Fig. 5b, c) and co-immunoprecipitation (co-IP) (Fig. 5d) assays to determine the physical interaction between MED15 and FOXA1. We performed a co-IP experiment in which the cells were treated with or without TGFβ to evaluate whether MED15 T603 phosphorylation affects this interaction. Notably, we observed that TGFβ-induced MED15 T603 phosphorylation apparently decreased the interaction between MED15 and FOXA1, whereas dephosphorylated MED15 T603A seemed to enhance this interaction (Fig. 5e). Importantly, the anti-FOXA1 antibody hardly pulled down any T603 phosphorylated form of MED15 (Fig. 5e), suggesting that MED15 T603 phosphorylation is indeed a crucial switch that modulates the interaction between MED15 and FOXA1.

FOXA1 is a key member of the forkhead family of winged-helix transcription factors; it was initially reported as a pioneer transcription factor that regulates liver organogenesis^30^ and has been suggested as a transcription repressor of breast cancer proliferation and metastasis^31^. We explored the genome-wide association between FOXA1 and MED15 T603 phosphorylation by performing ChIP-seq analyses of FOXA1 and Pol II in WT and MED15 T603A mutant HaCaT cells after TGFβ treatment for 3 h. We first analyzed the genome-wide occupancy of FOXA1 and Pol II and found that the MED15 T603A mutant did not alter the overall chromatin binding of FOXA1 and Pol II (Fig. 5f). Because the interaction between MED15 and FOXA1 was modulated by T603 phosphorylation, we investigated whether the MED15 T603A mutant affected the binding of FOXA1 and Pol II to the 722 downstream target genes of MED15 pT603 via RNA-seq analysis (Fig. 2h). As shown in Fig. 5g, the expression of these MED15 pT603 downstream genes, which are related to aging and the SASP, was downregulated by the T603A mutant regardless of TGFβ treatment, whereas their expression tended to be upregulated by the T603D mutant. Interestingly, in MED15 T603A mutant cells, FOXA1 binding to these downstream genes was increased, but Pol II binding signals were decreased (Fig. 5h–l).

We further verified the interplay among FOXA1, MED15 and Pol II in target gene regulation. Representative ChIP-seq data were visualized at specific genomic loci occupied by FOXA1 and Pol II. In MED15 T603A mutant cells, we observed increased FOXA1 binding and reduced Pol II binding at genes encoding SASP factors such as IGF1R, OAS1 and CD47. RNA-seq track plots revealed that the transcription of these SASP factors changed accordingly with the corresponding MED15 T603 mutants (Fig. 5m). The MED15 T603A mutant increased FOXA1 binding to the IGF1R, OAS1, and CD47 genes while reducing Pol II binding, which was consistent with the RNA-seq data. These SASP genes were downregulated by the MED15 T603A mutant but upregulated by the MED15 T603D mutant (Fig. 5m).

These experiments indicate that MED15 T603 phosphorylation regulates senescence by modulating downstream FOXA1 target genes and that the T603A mutant increases the interaction with FOXA1 to suppress the expression of SASP genes and to alleviate cellular senescence.

### Med15 T604A mutant mice exhibit resistance to cognitive aging

**Fig. 6:**
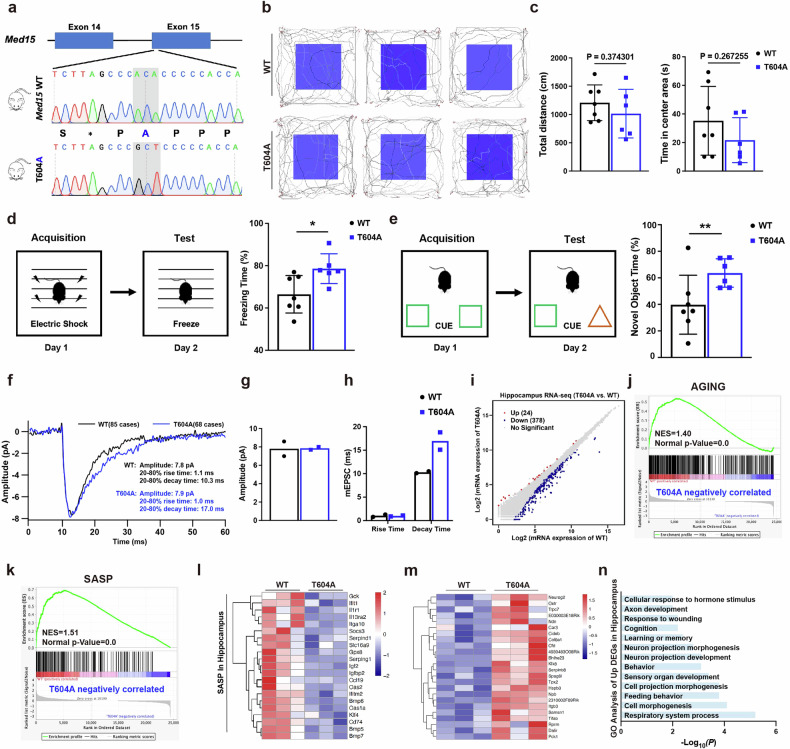
The Med15 T604A mutant prevents cognitive decline in aging mice. **a** Genotyping the endogenous Med15 T604A mutant in mice by PCR and Sanger sequencing. **b**, **c** Diagrams showing the results of the open-field test (**b**), including the total distance traveled and time spent in the center area (**c**), by old male WT (*n* = 7) and Med15 T604A mice (*n* = 6). The values are presented as means ± SDs. **d** Diagram showing the results of the fear-conditioning system test (left panel) and percentage of freezing time for old male WT (*n* = 7) and Med15 T604A mice (*n* = 6) (right panel). The values are presented as means ± SD. *P < 0.05. **e** Diagram showing the NOR test (left panel) and percentage of time spent exploring the novel object by old male WT (*n* = 7) and Med15 T604A mice (*n* = 6) in the NOR test (right panel). The values are presented as means ± SD. **P < 0.01. **f** mEPSCs recorded in CA1 hippocampal neurons of old male WT (85 cases) and Med15 T604A (68 cases) mice. **g** Amplitude of mEPSCs in CA1 hippocampal neurons of old male WT (85 cases) and Med15 T604A (68 cases) mice. **h** Rise time and decay time of mEPSCs in CA1 hippocampal neurons of old male WT (85 cases) and Med15 T604A (68 cases) mice. **i** Scatter plot of DEGs (FPKM, fold change ≥ 1.5, *P* < 0.05) identified by RNA-seq in the hippocampus of old (17–20 months) male WT and Med15 T604A mice (*n* = 3). **j**, **k** GSEA enrichment plots of the indicated gene sets. **l** Heatmaps of DEGs associated with SASP genes in the hippocampus of aged mice. **m** Heatmaps of DEGs upregulated by Med15 T604A in the hippocampus of aged mice. **n** GO analysis of DEGs upregulated by Med15 T604A in the hippocampus of aged mice.

Due to the crucial role of MED15 T603 phosphorylation in cellular senescence and the SASP, we wanted to test whether it plays a role in organismal aging. Conserved in humans and mice, MED15 T603 corresponds to the phosphosite of Med15 T604, in mice (Supplementary Fig. S4). To further characterize this phosphosite in mice, we generated a Med15 T604A (threonine to alanine) mutant in mice using the CRISPR-Cas9 technique (Fig. 6a). The Mendelian ratios of mice produced during breeding were not affected by the Med15 T604A mutant, and the mutant mice apparently developed normally without much difference from the WT mice, suggesting that this knock-in mutation did not disrupt the normal reproduction and development of the mice (Supplementary Table S1). The Med15 T604A mutant also had no influence on the bone density of aging mice (17–20 months) (Supplementary Fig. S5), implying that Med15 T604 phosphorylation may have a tissue-specific role in regulating aging.

Given that numerous neurodevelopment-related genes were upregulated in MED15 T603A mutant cells (Supplementary Fig. S2g), we examined whether Med15 T604 phosphorylation may regulate the neural and behavioral characteristics of aging mice (17–20 months). The open-field test revealed no difference in total movement distance and time spent in the central area between T604A mutant mice and WT mice (Fig. 6b, c), indicating that the basic athletic ability and anxiety level of aged Med15 T604A mice did not differ from those of WT mice. We thus performed additional behavioral analyses to assess whether Med15 T604A may impact cognitive ability. In the fear-conditioning system (FCS) test, on Day 1, the aging mice were trained in a fear-conditioned chamber and were shocked for an interval of 120 s for 10 min, and on Day 2, the aging mice were placed in the same chamber but were not exposed to shocks. Med15 T604A mice exhibited significantly longer freezing times than the control mice because they were better able to remember the fear of the shock (Fig. 6d). Similarly, in the novel object recognition (NOR) test, compared to aging WT mice, Med15 T604A mice spent more time exploring the replaced novel object (Fig. 6e). As demonstrated by the FCS test and NOR test, aging male Med15 T604A mice presented significantly improved memory and learning abilities.

Miniature excitatory postsynaptic currents (mEPSCs) were measured in the hippocampal CA1 neurons of aging WT and T604A mutant mice (20 months) to understand the electrophysiological changes associated with the changes in cognitive ability. As shown in Fig. 6f–h, Med15 T604A mice did not exhibit changes in the mEPSC amplitude or rise time, but the hippocampal neurons from mutant mouse brains displayed an increased decay time compared to the neurons from WT mice, suggesting that Med15 T604A neurons had a longer excitatory time than WT neurons, potentially contributing to the better behavioral performance of the aging mutant mice. We further performed RNA-seq analysis of hippocampus in aging mice, and found that Med15 T604A hippocampus showed decreased expression of various SASP genes and Med15 T604A was negatively correlated with aging and SASP in GSEA analysis (Fig. 6i–l; Supplementary Fig. S6a–c). Twenty-four genes that were upregulated by the T604A mutation were also associated with neurodevelopment (Fig. 6m, n), suggesting that the regulation of SASP and neurodevelopment-related gene expression by the Med15 T604A mutant confers resistance to hippocampal aging and behavioral deterioration.

**Fig. 7:**
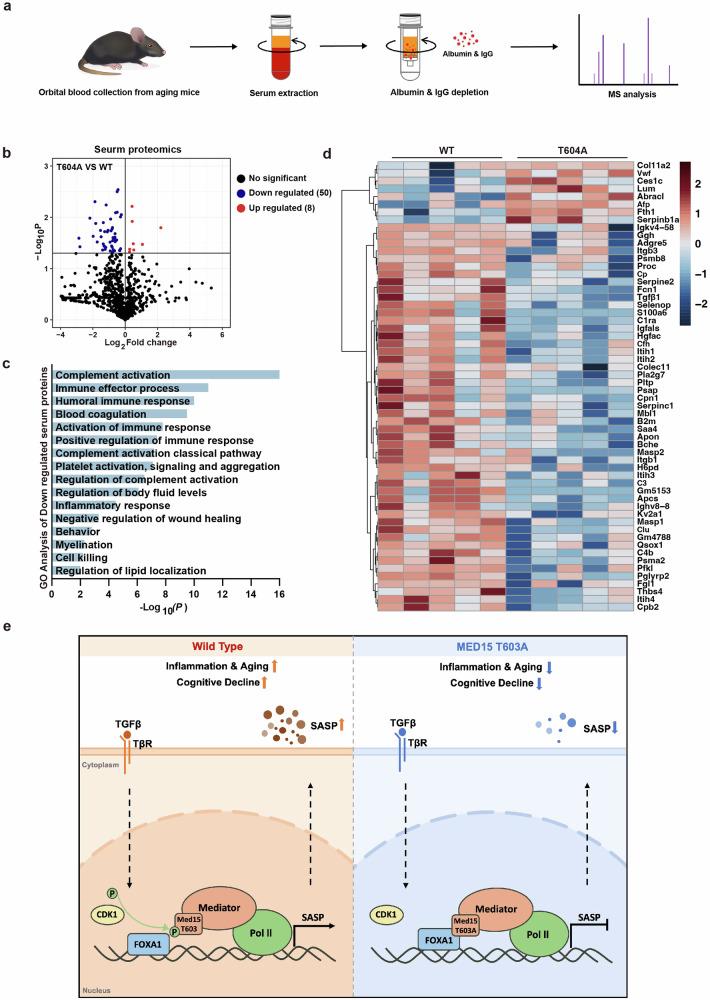
The T604A mutation relieves inflammation in the serum of aging mice. **a** Workflow of the serum proteomic assay. **b** Volcano plot of differential alterations in serum protein levels in old male WT and Med15 T604A mutant mice (*n* = 5). **c** GO analysis of serum proteins downregulated by Med15 T604A in aging mice (*n* = 5). **d** Heatmap of differential alterations in serum protein levels in WT and Med15 T604A mutant aging mice (*n* = 5). **e** Model of the regulatory function of MED15 T603 phosphorylation in aging. MED15 T603 phosphorylation is positively related to aging and the SASP. TGFβ increases the phosphorylation of this site by the kinase CDK1. Phosphorylated MED15 T603 aggravates cellular senescence through upregulation of the SASP. Dephosphorylated MED15 T603 functions as a brake to restrain cellular senescence by intensifying FOXA1-mediated inhibition of SASP genes. The Med15 T604A mutant prevents cognitive decline and serum inflammation in aging mice.

Considering the possible systemic effects of SASP factors across tissues, we performed proteomic profiling of serum from aging WT and T604A mice (17–20 months) (Fig. 7a). Compared to those in WT mouse serum, the levels of 8 proteins were increased and 50 proteins were decreased in T604A mouse serum (Fig. 7b). The GO analysis of the decreased proteins revealed an association with inflammatory responses (Fig. 7c). The levels of SASP factors such as Tgfβ1, Serpine1, Serpinc1 and Itgb1 were significantly reduced in the serum of Med15 T604A mutant mice, suggesting that the inflammation level in the aging mouse circulation was alleviated in Med15 T604A mice (Fig. 7d), which contributed to the anti-aging effect of the Med15 T604A mutant across tissues. Overall, these results indicate that Med15 T604A does not affect reproduction or normal development of mice but results in reduced SASP levels in the hippocampus and serum; therefore, Med15 T604A mice are resistant to cognitive decline.

## Discussion

In this study, we first discovered an evolutionarily conserved phosphosite in Mediator MED15 via MED15 IP-MS and bioinformatic analysis (Fig. 1a–d), and its biological function and regulatory mechanism are previously unknown. We then knocked in a dephosphorylated mutation (MED15 T603A) in HaCaT cells to explore the role of MED15 T603 phosphorylation in TGFβ signaling and aging (Fig. 1e). Alleviation of cell growth inhibition by TGFβ treatment was observed in MED15 T603A mutant cells, accompanied by decreased expression of p21, a classical cellular senescence marker (Fig. 1f–i). Consistent with the decrease in p21 expression in T603A mutant cells, cellular senescence was obviously relieved after TGFβ treatment compared to that in WT cells, and the levels of SASP factors were also dramatically reduced by the T603A mutant (Fig. 2a–d). These data suggest that MED15 T603 phosphorylation is intimately related to cellular senescence. A further transcriptome analysis revealed that the genes downstream of MED15 pT603 are associated with the inflammatory response and cellular senescence and that dephosphorylation of this site confers resistance to aging and SASP (Fig. 2e–j). Conversely, aggravated cell cycle arrest and senescence were observed in MED15 T603 phosphorylation-mimicking mutant cells (T603D), aging and SASP genes were activated by the T603D mutation (Fig. 3a–k). Together, these findings indicate a connection between MED15 T603 phosphorylation and aging.

Having developed an antibody that specifically recognizes MED15 T603 phosphorylation, we found that CDK1 is the upstream kinase of this phosphosite, and that knocking down or inhibiting CDK1 reduces p21 and SASP expression through MED15 T603 dephosphorylation (Fig. 4a–j). CDK1 expression is correlated with aging in colorectal cancer patients^32^, CDK1 activity is essential for efficient p21 production and cellular senescence^33^, and its specific inhibitor Ro3306 has been shown to protect against postovulatory aging^34^; however, the underlying mechanisms through which CDK1 regulates aging is unclear. Our study revealed that CDK1 may regulate tissue aging through catalyzing the MED15 T603 phosphorylation, providing a novel mechanism for understanding how CDK1 regulates p21 and its related aging processes.

In efforts to understand the molecular mechanism underlying the regulation of MED15 T603 phosphorylation during the SASP and aging, we found that FOXA1 may play a critical role via a motif analysis of MED15 pT603-target gene sets. Specifically, the TGFβ-induced MED15 T603 phosphorylation functions as a brake to restrain its interaction with FOXA1, whereas the MED15 T603A dephosphorylation-mimic mutant significantly increased its interaction with FOXA1 to reinforce the repression of MED15 pT603 downstream genes, such as the SASP factors IGF1R, OAS1 and CD47 (Fig. 5a–m). It is intriguing that previous studies have found that FOXA1 may play an either positive or negative role in transcription regulation. A previous work revealed that FOXA1 functions as a chromatin regulator to modulate gene expression. In detail, FOXA1 intrinsically forms submicron-sized condensates through its N- and C-terminal intrinsically disordered regions to unpack condensed chromatin and promote gene expression, suggesting a transcriptional activator function of FOXA1^35^. On the other hand, it was reported that the *O*-linked β-N-acetylglucosamine modification (*O*-GlcNAcylation) of FOXA1 shapes its interactome, and triggers the recruitment of the transcriptional repressor MECP2 to inhibit the expression of adhesion-related genes, indicating a repressor role for FOXA1 in gene regulation^31^. In our study, to understand the novel mechanism of FOXA1 regulation in aging, we uncovered that FOXA1 acts as a repressive transcription factor to collaborate with the T603-dephosphorylated MED15 for SASP gene inhibition and to relieve senescence. Considering that MECP2 mediates transcriptional silencing at methylated CpG dinucleotide (CG) regions^36^, and the Mediator subunits, such as MED1^37,38^, MED12^39^, and MED23^23^, are also involved in the process of remodeling the chromatin structure and regulating gene expression through long-distance chromatin interactions, we postulate that MECP2, HDACs and/or other epigenetic regulators may be involved in the transcriptional regulation of the MED15 pT603 downstream genes and the aging process. Future studies are needed to address how exactly multiple epigenetic regulators orchestrate the sophisticated aging-related gene regulation.

Remarkably, the anti-aging effects on cognitive decline were observed in the generated Med15 T604A mice, as evidenced by their improved learning and memory in behavioral experiments. Electrophysiological experiments also revealed longer excitatory times in T604A neurons, potentially contributing to reduced behavioral deterioration. Consistent with previous data obtained from cell lines, the transcriptomic analysis of the hippocampus revealed decreased SASP gene expression and increased neurodevelopment-related gene expression in T604A mutant mice (Fig. 6a–n). Previous studies have shown that neural inflammation has a strong effect on the synaptic signal transduction of hippocampal neurons. For example, an increase in IL1β levels can weaken the strength of synaptic transmission between hippocampal CA1 neurons, a process implicated in the progression of neurodegenerative diseases such as multiple sclerosis^40–42^. Our study revealed that aging Med15 T604A mutant mice presented reduced SASP gene expression, which in turn inhibited hippocampal inflammation, alleviating the degeneration of synaptic signal transmission between hippocampal CA1 neurons and ultimately mitigating memory and cognitive decline in aging mice. We therefore demonstrated that a novel phospho-switch in the MED15 controls neural inflammation and cognition ability.

Furthermore, several studies have reported that the plasma level of TGFβ increases significantly in aging humans^43,44^, which reinforces the SASP in human fibroblasts^45^. Due to the paracrine effects of the SASP across tissues, we performed serum proteomic analyses to investigate whether the levels of SASP factors in the fluid circulation were affected by T604A mutant. Indeed, SASP factors, such as Tgfβ1, Serpine1, Serpinc1, Itgb1 and Itgbc, were reduced in T604A mutant mouse serum, indicating a lower level of inflammation in the circulation, which is beneficial for the rejuvenation of aging tissues (Fig. 7a–d). However, the spleen weight and immune cell proportion were not affected by the T604A mutation in aging mice (Supplementary Fig. S7), suggesting that this mutation may specifically alleviate inflammation across tissues without disrupting immune system development.

In summary, our study demonstrated a novel molecular mechanism to regulate aging. MED15 T603 phosphorylation is a novel phospho-target that modulates aging and the SASP across organs. These findings further elucidate the biological role and transcriptional regulation of the Mediator subunit MED15 T603 phosphorylation site, providing valuable insights into the mechanisms modulating the SASP and suggesting possible ways to achieve healthy aging (Fig. 7e). Small-molecule drugs that target MED15 pT603 may have the potential to ameliorate age-related diseases, such as Alzheimer’s disease, Parkinson’s disease, and obesity.

## Materials and methods

### Cell culture and targeted genome editing

Human immortalized epidermal HaCaT cells and breast cancer MCF7 cells were obtained from the Cell Resource Center of the Chinese Academy of Sciences. Cells were cultured in high-glucose Dulbecco’s modified Eagle’s medium (DMEM, HyClone, Logan, UT, USA) containing 10% fetal bovine serum (FBS). sgRNAs targeting the MED15 T603 site were cloned and inserted into the CRISPR/Cas9 plasmid vector pX330, which was co-transfected into cells with a 3500 bp DNA template cloned and inserted into the plasmid vector pEGFP-N1 used for homologous directed DNA repair. The cells were sorted via FACS and validated by Sanger sequencing.

### Western blotting

For western blotting, the cells were sonicated in lysis buffer (50 mM Tris‑HCl, pH 7.5, containing 150 mM NaCl, 0.1 mM EDTA, 1% Triton X‑100, 1 μg/mL aprotinin, 10 μg/mL leupeptin, and 1 mM PMSF) and centrifuged at 20,000× *g* for 10 min. The supernatants were subjected to SDS-PAGE, followed by western blot analyses with the indicated antibodies. Enhanced Chemiluminescence Plus Western Blotting Detection Kits (Bio-Rad) and a luminescent image analyzer (Tanon, Shanghai, China) were used to visualize the protein bands according to the manufacturer’s instructions. The following antibodies were used for western blot: anti-MED1 (#A300-793A, Bethyl), anti-MED12 (#A300-774A, Bethyl), anti-MED15 (#ab181158, Abcam), anti-MED23 (#ab200351, Abcam), anti-CDK1 (#A0220, ABclonal), anti-p21 (#A1483, ABclonal), anti-IL1β (#A16288, ABclonal), anti-β-Actin (#AC006, ABclonal), anti-GAPDH (#AC054, ABclonal), anti-HA (#AE036, ABclonal), anti-FLAG (#AE092, ABclonal), anti-pSMAD2(S465/467) (#18338, Cell Signaling Technology), anti-SMAD2 (#5339, Cell Signaling Technology), anti-pSMAD3(S423/425) (#9520, Cell Signaling Technology), anti-SMAD3 (#9513, Cell Signaling Technology), anti-pERK (Thr202/Tyr204) (#9101, Cell Signaling Technology), anti-ERK (#9102, Cell Signaling Technology), anti-FOXA1 (#A15278, ABclonal).

### Cell proliferation assay

Cell proliferation assays were performed using cell counting kit-8 (CCK-8) reagent (Dojindo Laboratories, Kumamoto, Japan). Briefly, differently treated cells were transferred into 96-well plates at a density of 4 × 10^3^ cells in 100 µL of growth medium per well and examined at different time points. At each time point, 10 µL of CCK-8 reagent was added to each well and incubated at 37 °C for 1 h. The absorbance at 450 nm was measured using a multimode microplate reader (BioTek, Vermont, USA).

### Cell cycle analysis

Differently treated cells were washed with phosphate-buffered saline (PBS). The cells were resuspended in PBS containing propidium iodide (10 mg/mL), Triton X-100 (3%) (Sigma-Aldrich) and RNase A (50 µg/mL) and incubated in the dark for 15 min. The fractions of viable cells in G1, S, and G2 phases of the cell cycle were measured with a FACStar flow cytometer (BD Biosciences, San Jose, CA, USA). Flow cytometry was performed as previously described^46^.

### IP-MS and analysis

We employed a comprehensive IP-MS workflow using a custom-engineered 293T cell line to delineate the interactome and posttranslational modifications of the MED15 protein, particularly focusing on phosphorylation events. This cell line was genetically engineered via CRISPR/Cas9-mediated knock-in to introduce an N-terminal HA tag to the MED15 gene, enabling specific enrichment of the tagged protein and its binding partners. The cells were grown in DMEM supplemented with 10% FBS and 1% penicillin-streptomycin until they reached ~80% confluence. Nuclear extracts were prepared, followed by immunoprecipitation with magnetic beads covalently conjugated with an anti-HA antibody to selectively capture the HA-tagged MED15 protein and its associated interactome. After elution of the captured proteins, trypsin digestion was performed, and the resulting peptides were analyzed on a Fusion Lumos Tribrid mass spectrometer coupled with a nanoACQUITY UPLC system. The MS data were acquired in a data-dependent acquisition mode optimized for phosphopeptide identification. Precursor ions were isolated in the quadrupole, fragmented by higher-energy collisional dissociation (HCD), and detected in the Orbitrap analyzer with a resolution of 120,000 at m/z 200. The raw data files were processed using MaxQuant software (version 1.6.2.10) for peptide identification and quantification, referencing the UniProt human proteome database. In addition to standard protein identification parameters, we specifically searched for phosphorylation sites on serine, threonine, and tyrosine residues using MaxQuant’s integrated Andromeda search engine. The search parameters included up to two missed cleavages, a maximum peptide length of 25 amino acids, and a minimum peptide length of 7 amino acids. Carbamidomethylation of cysteines was set as a fixed modification, whereas oxidation of methionines and N-terminal protein acetylation were set as variable modifications. The identified peptides and proteins were filtered at a 1% false discovery rate (FDR) using a decoy database strategy.

### SA-β-gal staining

SA-β-gal staining was performed using a senescence β-galactosidase staining kit (#9860, Cell Signaling Technology). Briefly, cultured cells were fixed for 15 min at room temperature in 1× fixative solution. Fixed cells were stained with SA-β-gal staining solution at 37 °C overnight in a dry incubator (without CO_2_), and the number of SA-β-gal-positive cells was then calculated.

### qRT-PCR and RNA-seq

Total RNA was extracted from cells lysed with TRIzol. One microgram of total RNA was reverse transcribed (#R047A, TaKaRa-Bio, Dalian, China) to cDNA for qRT-PCR. We used a commercial RNA library kit to prepare the RNA-seq library according to the manufacturer’s specifications (VAHTS® Universal V8 RNA-seq LibraryPrep Kit) for Illumina. Briefly, mRNA was captured with mRNA capture beads before fragmentation. The RNA fragments were reverse transcribed and subjected to library amplification for Illumina (Novaseq 6000) sequencing. Primer sequences are listed in Supplementary Table S2.

### RNAi

Small interfering RNAs (siRNAs) used for the knockdown of CDK1 and nonspecific negative control siRNAs were designed and synthesized by GenePharma (Suzhou, China). The cells were seeded in a 6-well cell culture plate and cultured in growth medium on the day before transfection. The siRNAs were transfected using Lipofectamine RNAiMAX Reagent (Invitrogen, CA, USA) according to the manufacturer’s instructions. The cells were harvested for qRT-PCR 48 h later. The sequences of siRNAs are listed in Supplementary Table S3.

### ChIP assay and ChIP-seq

ChIP-seq assays were conducted as previously described^23^. Briefly, the cells were crosslinked with 0.9% formaldehyde for 9 min at room temperature, and the crosslinking was quenched with glycine at a final concentration of 125 μM. After 5 min, the cells were washed twice with ice-cold PBS and collected with a cell scraper. The cell pellets were lysed and sonicated in lysis buffer (1% SDS, 50 mM Tris-HCl, pH 7.4, and 10 mM EDTA), and the chromatin was fragmented into 200–500 bp fragments using a COVARIS S220 sonicator. The lysates were then diluted with dilution buffer (0.01% SDS, 1.1% Triton X-100, 16.7 mM Tris-HCl, pH 8.0, and 1.2 mM EDTA), and immunoprecipitation was performed overnight at 4 °C with 2–4 μg of antibodies against FOXA1 (#A15278, ABclonal) or polymerase II (Pol II, #sc-899, Santa Cruz) and chromatin corresponding to 1 × 10^6^ cells. The next day, the IP samples were incubated with Protein G Dynabeads for 2 h at 4 °C, and the beads were washed with low-salt buffer (0.1% SDS, 1% Triton X-100, 1 mM EDTA, 20 mM HEPES, pH 7.9, 150 mM NaCl, and 0.1% deoxycholate), high-salt buffer (0.1% SDS, 1% Triton, 1 mM EDTA, 50 mM HEPES, pH 7.9, 500 mM NaCl, and 0.1% deoxycholate), LiCl buffer (0.25 M LiCl, 0.5% NP40, 0.5% deoxycholate, 1 mM EDTA, and 20 mM Tris-HCl, pH 8.0) and TE buffer (10 mM Tris-HCl, pH 8.0 and 1 mM EDTA) before elution. The cross-links in the eluates were reversed at 65 °C, and the DNA was purified. The DNA was subjected to library construction (NEBNext Ultra II DNA Library Prep Kit, NEB, E7645B, USA) for Illumina sequencing.

### ChIP-seq and RNA-seq analyses

ChIP-seq and RNA-seq raw reads were obtained from an Illumina NovaSeq X Plus Series PE150 sequence analyzer. The raw RNA-seq reads were obtained from an Illumina NovaSeq 6000 PE150 sequence analyzer. We utilized FastQC (v0.1.0) for raw data quality summary. Cutadapt was used to remove sequencing adapters and trim poor-quality bases, retaining only reads with a quality score greater than 20 and discarding reads shorter than 20 bp. For RNA-seq, the filtered clean reads were mapped to the human reference genome hg19 or mouse reference genome mm10 with HISAT2, and gene quantification and the differential expression analysis were performed with HTSeq count and DEseq2. GSEA was performed using the GSEA software (v4.0.3). RNA-seq data were analyzed against predefined gene sets, including “AGING” and “SASP”, by integrating data from the Aging Atlas database and published literature, complete gene list has been provided in Supplementary Tables S4, S5. The analysis was conducted with default parameters, and significance was assessed based on FDR (< 0.25) and normalized enrichment score (NES). For ChIP-seq, clean reads were aligned to the human reference genome hg19 with Bowtie2 and converted to bigwig files for visualization by bamCoverage. Peak calling was conducted via MACS2^47^ with two replicates, and peaks were annotated using the R packages ChIPpeakAnno^48^ and ChIPseeker^49^. The ChIP-seq signal intensity was calculated and normalized to the CPM, and *P* values were calculated using two-sample Student’s *t*-test or the K–S test. Finally, all heatmaps from the ChIP-seq data and density profiles were generated with DeepTools.

### Mice

All procedures involving mice were approved by the Fudan University Animal Care and Use Committee. The mice were housed in the animal facility and had free access to standard rodent chow and water. Med15 T604A mutant mice were generated by Cyagen Biosciences (Suzhou, China). The genotypes of the mice were validated by Sanger sequencing.

### Open-field test

Locomotor activity and anxiety-like behavior of the mice were assessed in sound-insulated, rectangular activity chambers (Omnitech SuperFlex). Each mouse was placed in a 40 cm × 40 cm × 30 cm box and allowed to explore freely for 10 min. The behaviors, including total distance traveled, number of times the mouse entered the center area, and time spent in the center area, were recorded and evaluated for 10 min with Fusion v6 for superflex (Omnitech Electronics, Inc.).

### Contextual fear conditioning test

On Day 1, each mouse was placed in a fear conditioning chamber (Actimetrics ACT-100A), and five 0.6 mA shocks spaced 120 s apart were administered at 2 min, 4 min, 6 min, 8 min and 10 min of the task. On Day 2, each mouse was placed in the fear conditioning chamber containing the same context but with no administration of foot shocks. Freezing was analyzed for 1–3 min. Freezing was measured using Freeze Frame software (Actimetrics, v5).

### NOR test

On Day 1 (the training phase), two identical objects were placed in the habituated arena, and the mice were allowed to explore for 10 min. On Day 2 (the testing phase), one object was replaced with a novel object, and the mice were allowed to explore for 10 min. The time spent exploring each object was quantified using EthoVisionXT (Noldus Information Technology, The Netherlands). Half of the mice were exposed to object A as their novel object and half to object B to control for any inherent object preference. We determined the percentage of time spent exploring the novel object as follows: (time spent exploring the novel object)/(time spent exploring the trained object + time spent exploring the novel object) × 100.

### Electrophysiology

Male WT and Med15 T604A mutant mice (20 months old) were decapitated, and tissue blocks containing the hippocampus were placed in a low-Ca^2+^ artificial cerebrospinal fluid (ACSF) solution (125 mmol/L NaCl, 25 mmol/L NaHCO_3_, 3 mmol/L myo-inositol, 2 mmol/L Na-pyruvate, 2.5 mmol/L KCl, 1.25 mmol/L NaH_2_PO_4_, 0.4 mmol/L ascorbic acid, 25 mmol/L glucose, 3 mmol/L MgCl_2_, and 0.05 mmol/L CaCl_2_). The brain slices (~350-μm thick) were sectioned using a vibratome (VT 1200 s, Leica, Germany) and incubated in normal ACSF containing 2 mmol/L CaCl_2_ at 37 °C for 30–40 min before the experiments were performed. All electrophysiological recordings were made at room temperature (22–24 °C). Voltage-clamp recordings of mEPSCs were obtained using an EPC-10 amplifier (HEKA, Lambrecht, Germany). The pipette (2–3 mΩ) solution contained 125 mmol/L K-gluconate, 20 mmol/L KCl, 4 mmol/L Mg-ATP, 10 mmol/L Na_2_-phosphocreatine, 0.3 mmol/L GTP, 10 mmol/L HEPES, and 0.5 mmol/L EGTA (pH 7.2, adjusted with KOH). The extracellular solution was similar to the ACSF except that it contained 2 mmol/L CaCl_2_. The series resistance (< 10 mΩ) was compensated by 95% (lag 10 μs) throughout the experiment. The mEPSCs were recorded at a holding membrane potential of –80 mV in the presence of 0.5 μmol/L tetrodotoxin. The Mini Analysis Program (version 6.07; Synaptosoft, USA) was used to analyze the mEPSCs.

### Serum proteomics

The serum was collected from male WT and Med15 T604A mutant mice (17–20 months). The mouse serum protein solution was vacuum dried after removal of the IgGs by an Albumin/IgG Depletion Kit (Beyotime, P2295M). Then, the sample was resuspended in SDS-PAGE loading buffer and boiled. The SP3 digestion method was adapted. LC-MS analysis was performed using a nanoelute2 system coupled to an TimsTOF HT mass spectrometer (Bruker Scientific, Bremen, Germany). A one-column system was adopted for all analyses. Samples were analyzed on a home-made C18 analytical column (75 µm i.d. × 25 cm, ReproSil-Pur 120 C18-AQ, 1.9 µm (Dr. Maisch GmbH, Germany)). The mobile phases consisted of Solution A (0.1% formic acid in 2% ACN) and Solution B (0.1% formic acid in 80% ACN). The peptides were separated within 60 min gradients (6%–28% B in 43 min, 28%–80% B in 7 min, 100% B for 10 min, flow rate of 300 nL/min). After ionization, peptides were measured using timsTOF HT with PASEF window scheme in 100–1700 m/z and combined with a mobility range from 0.75 to 1.5 1/K0. Ramp and accumulation time were set to 100 ms. Data were processed with Mus_musculus database (55428 entries, download in 20200321) using FragPipe (v22.0) use the LFQ-MBR workflow.

### Flow cytometry

Single-cell suspensions of the spleen were prepared, and red blood cells were lysed before staining. The cell suspensions were divided into two parts. One part was stained with antibodies specific for mouse CD45 (APC), CD3 (APC-Cy7), CD4 (FITC), CD8a (PE), CD19 (BV421), and NK1.1 (PE-Cy7). The other part was stained with antibodies against mouse CD45 (APC), MHCII (AF700), F4/80 (BV421), CD11b (PerCP-Cy5.5), CD11c (FITC), and Ly6G (PE). After being incubated in the dark for 20 min, the cells were washed three times and then analyzed via FACS. The cells were infected with VSV-GFP, and the level of green fluorescence was detected using a FACS cytometer (Fortessa, BD). The data were analyzed with FlowJo software (TreeStar).

### Statistical analyses

Statistical analyses were performed using GraphPad Prism software. The data are presented as means ± SD, with *n* indicating the number of independent experiments. Differences among the groups were compared via Student’s *t*-test. Differences were considered significant when *P* value was < 0.05. *P* values are depicted in figures with 1–3 asterisks (*P < 0.05, **P < 0.01 and ***P < 0.001).

## Supplementary information

Supplementary information

## Data Availability

All data supporting the findings of this study are available within the paper and its Supplementary information. The RNA-seq data generated in this study has been deposited in GEO dataset with accession number GSE285138. The ChIP-seq data generated in this study has been deposited in GEO dataset with accession number GSE285137. Any information required to reanalyze the data reported in this paper is available from the corresponding author upon request.
