# Supplementary material for: A phosphorylation switch in the Mediator MED15 controls cellular senescence and cognitive decline
Source: Cell Discov. 2025 Aug 19;11:69. doi: 10.1038/s41421-025-00820-1 (PMC12365249; doi:10.1038/s41421-025-00820-1)
Supplement: Supplementary file 1 — Supplementary information [file 41421_2025_820_MOESM1_ESM.pdf]

Supplementary Information

A phosphorylation switch in the Mediator MED15 controls cellular senescence and cognitive decline

Haozheng Li[1,4], Yuanming Zheng[1,4], Chunlei Yuan[1,4], Jiayi Wang[1], Xiaying Zhao[1], Ming Yang[1,2], Defei Xiong[1], Yenan Yang[1,2], Yunpeng Dai[3], Yiming Gao[1], Yuqi Wang[1], Lei Xue[1], Gang Wang[1,5]*

[1] State Key Laboratory of Genetics and Development of Complex Phenotypes, School of Life Sciences and Zhongshan Hospital, Fudan University, Shanghai, 200438, China
[2] School of Biological Sciences, The University of Hong Kong, Hong Kong, 999077, China
[3] School of Life Sciences, Westlake University, Hangzhou 310030, Zhejiang, China
[4] These authors contributed equally
[5] Lead contact
*Correspondence: gwang_fd@fudan.edu.cn

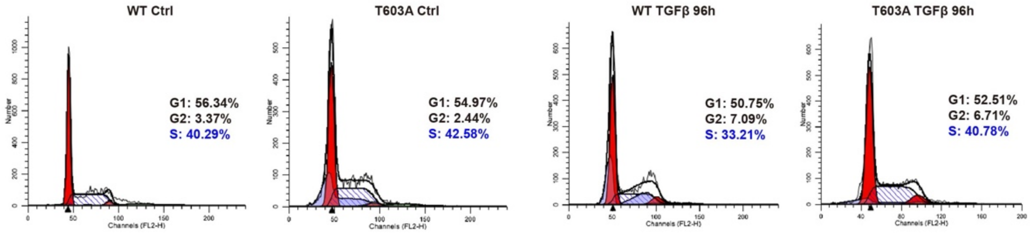

Figure S1. The MED15 T603A mutant confers resistance to TGFβ-induced cell cycle arrest. Flow cytometry analysis of the cell cycle distribution of WT and MED15 T603A mutant HaCaT cells treated without or with TGFβ.

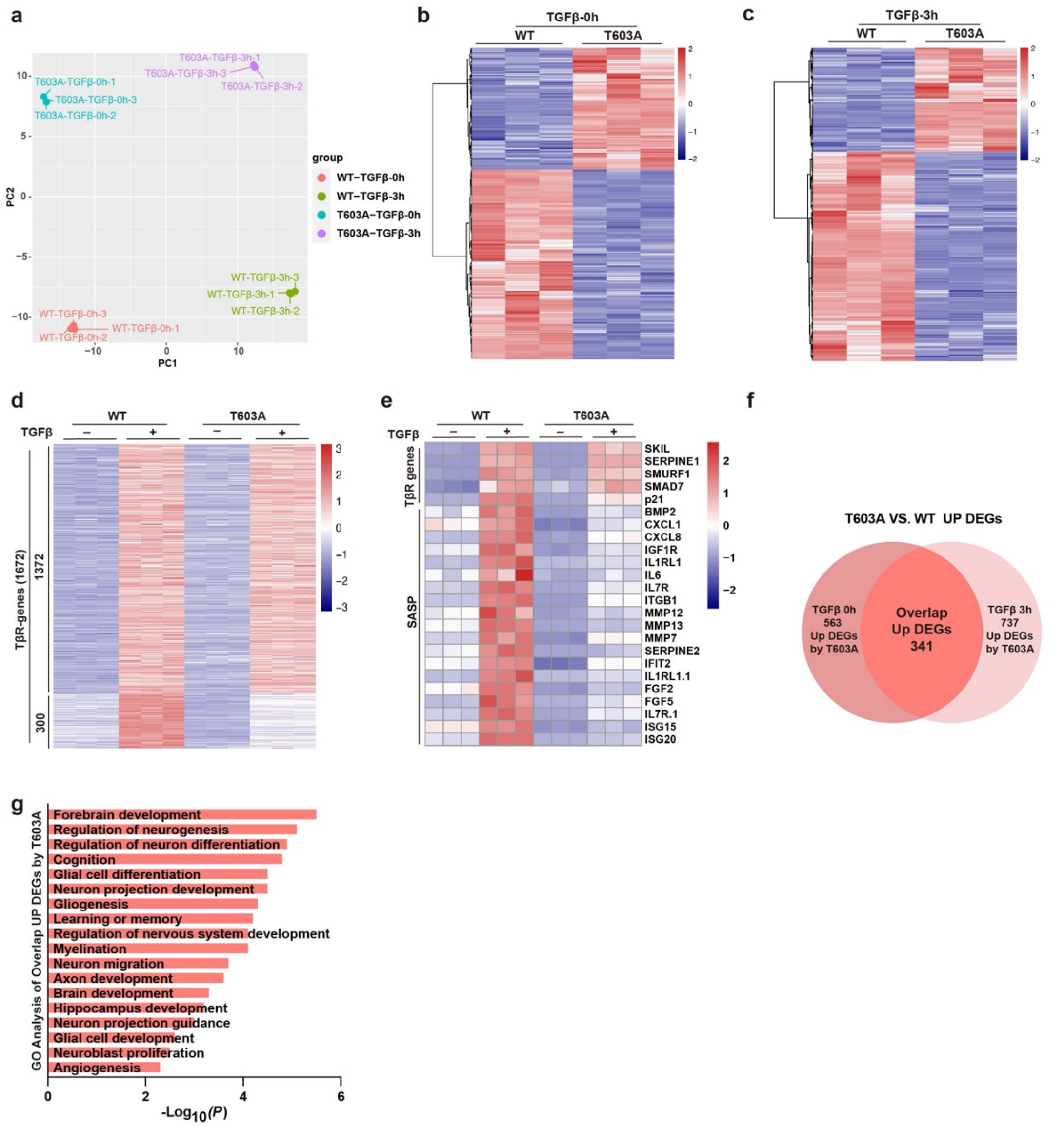

Figure S2. The MED15 T603A mutation relieves SASP

(a) Principal component analysis (PCA) of the transcriptomes of WT and MED15 T603A mutant HaCaT cells treated without or with TGFβ.

(b) Heatmap of differentially expressed genes (DEGs) identified by RNA-seq in WT and MED15 T603A mutant HaCaT cells in the steady state.

(c) Heatmap of DEGs identified by RNA-seq in WT and MED15 T603A mutant HaCaT cells after 3 h of TGFβ treatment.

(d) Heatmap of the TGFβ-responsive genes (TβR genes) identified by RNA-seq in WT and MED15 T603A mutant HaCaT cells treated without or with TGFβ.

(e) Heatmap of the SASP genes and TβR genes identified by RNA-seq in WT and MED15 T603A mutant HaCaT cells treated without or with TGFβ.

(f) Venn diagram showing the 341 overlapping genes among the upregulated DEGs identified by RNA-seq in the MED15 T603A mutant HaCaT cells.

(g) GO analysis of the 341 overlapping DEGs associated with the MED15 T603A mutation in HaCaT cells.

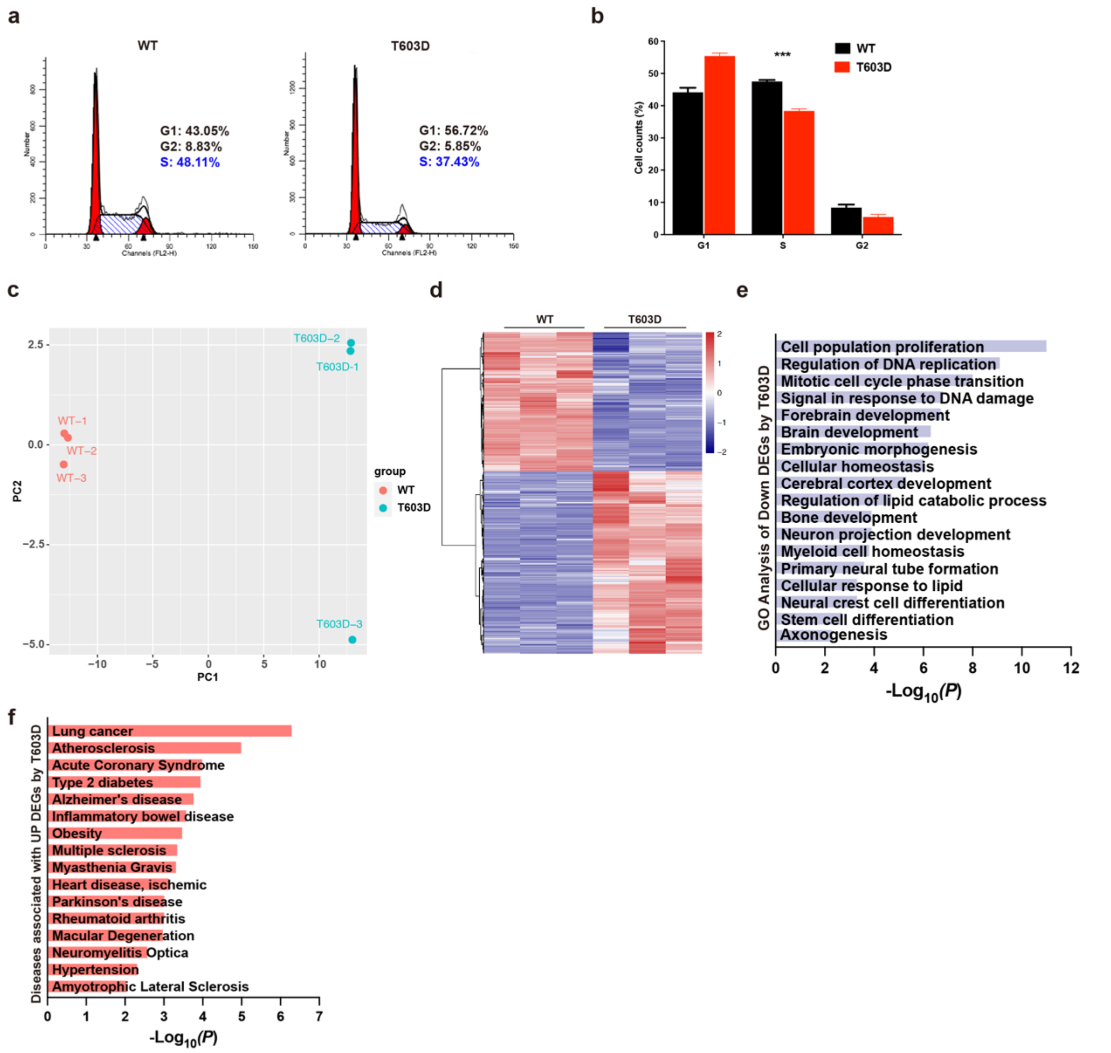

Figure S3. The MED15 T603D mutation exacerbates the SASP.

(a) Flow cytometry assays and statistical analysis (b) were used to analyze the cell cycle distribution of WT and MED15 T603D mutant MCF7 cells. The values are presented as the means ± SDs. ***P < 0.001.

(c) PCA of the transcriptomes of WT and MED15 T603D mutant MCF7 cells.

(d) Heatmap of DEGs identified by RNA-seq in WT and MED15 T603D mutant MCF7 cells.

(e) GO analysis of DEGs downregulated by the MED15 T603D mutation.

(f) Diseases predicted to be associated with upregulated genes by the MED15 T603D mutation.

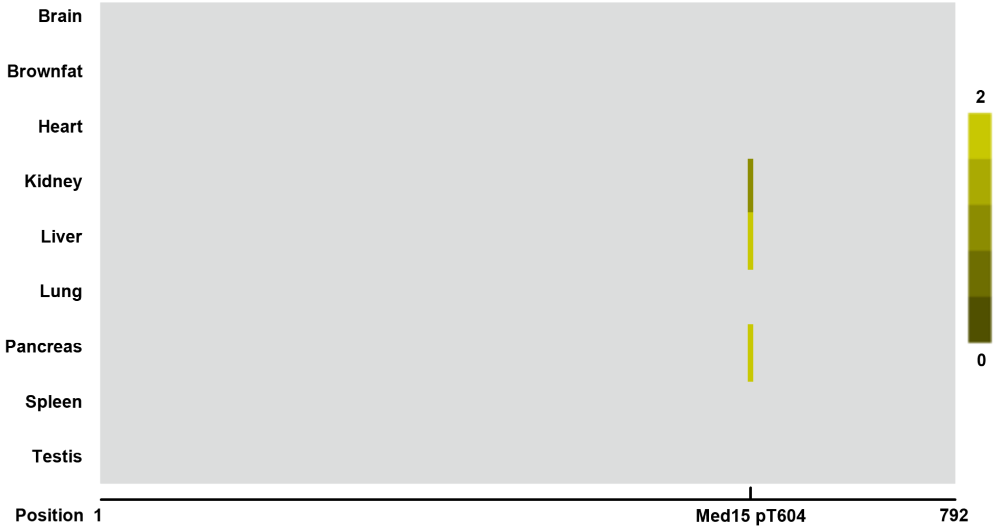

Figure S4. pT604 is the only known phosphosite of Med15 in mice.

Phosphosite analysis of Med15 in mice via the PhosphoMouse database.

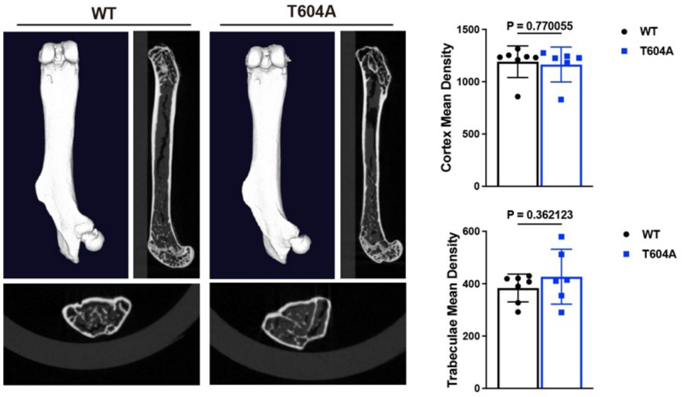

Figure S5. Bone density in aging WT and Med15 T604A mutant mice.

Representative μCT images of tibias from old male WT mice (n=7) and Med15 T604A (n=6) mutant mice.

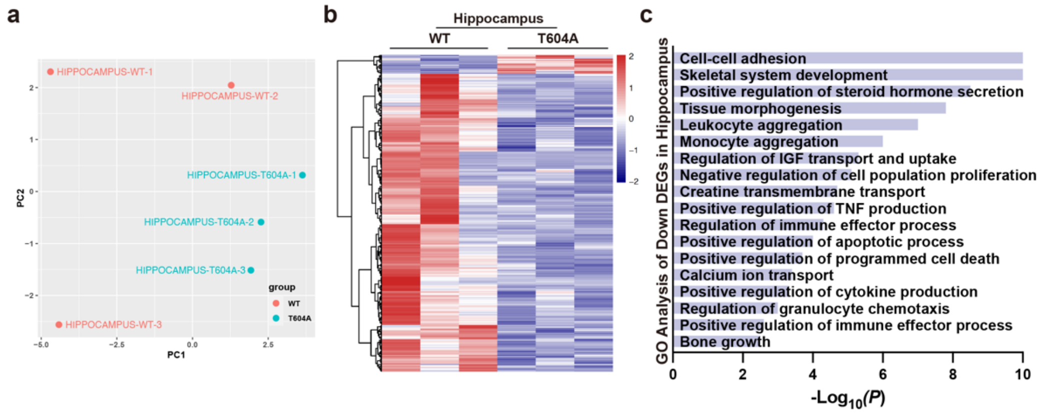

Figure S6. The Med15 T604A mutant alleviates the SASP in the hippocampus of aging mice.

(a) PCA of the transcriptomes of the hippocampus of the old male WT and Med15 T604A mutant mice (n=3).

(b) Heatmap of DEGs identified by RNA-seq in the hippocampus of old male WT and Med15 T604A mutant mice (n=3).

(c) GO analysis of DEGs downregulated by the Med15 T604A mutation in the hippocampus of aging mice.

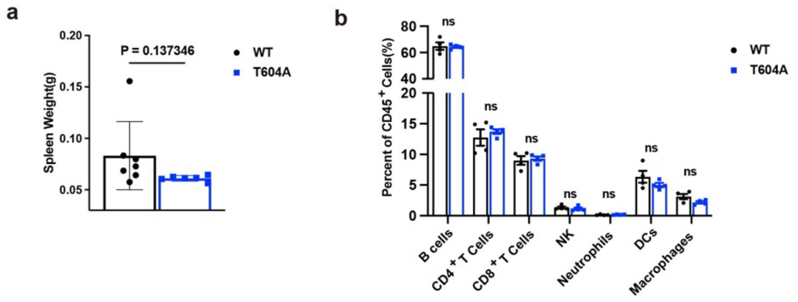

Figure S7. Proportion of splenic immune cells in aging WT and Med15 T604A mutant mice.

(a) Spleen weights of old male WT (n=7) and Med15 T604A mutant (n=6) mice.

(b) Proportions of splenic immune cells in old male WT and Med15 T604A mutant mice (n=3).

Table S1. Mendelian ratios of the outcome of Med15 T604A/+ mice breeding.

Genotype	Number of mice(%)
Med15 +/+	75(27.7%)
Med15 T604A/+	128(47.2%)
Med15 T604A/T604A	68(25.1%)
Total	271(100%)

Table S2. qRT-PCR primer sequences.

Gene	Forward	Reverse
ACTIN	GCACGGCATCGTCACCAACT	CATCTTCTCGCGGTTGGCCT
CDK1	GGGTCAGCTCGTTACTCAAC	TGACATGGGATGCTAGGCTT
CTGF	CTGCAGGCTAGAGAAGCAGAG	GATGCACTTTTTGCCCTTCT
CXCL1	CTGCTCCTGCTCCTGGTAG	TGGCTATGACTTCGGTTTGG
CXCL3	GCAGGGAATTCACCTCAAGA	GGTGCTCCCCTTGTTCAGTA
CXCL8	CAGCCTTCCTGATTTCTGC	ACTTCTCCACAACCCTCTGC
CXCL10	CCATTCTGATTTGCTGCCTTA	CCTTTCCTTGCTAACTGCTTTC
IL1α	TGGTAGTAGCAACCAACGGGA	ACTTTGATTGAGGGCGTCATTC
IL1β	ACTTTGATTGAGGGCGTCATTC	CGTTATCCCATGTGTCGAAGAA
IL6	CCTGAACCTTCCAAAGATGGC	TTCACCAGGCAAGTCTCCTCA
MMP1	GATGTGGAGTGCCTGATGTG	TGATGTCTGCTTGACCCTCA
MMP2	AGTGGATGATGCCTTTGCTC	GAGTCCGTCCTTACCGTCAA
MMP3	CGGTTCCGCCTGTCTCAAG	CGCCAAAAGTGCCTGTCTT
MMP12	TTGATGGCAAAGGTGGAATC	AGCAGAGAGGCGAAATGTGT
MMP13	CTTTGGCTTAGAGGTGACTGG	AGGCACTCCACA TCTTGGTTT
p21	TTAGCAGCGGAACAAGGAGT	GCCGAGAGAAAACAGTCCAG
PAI1	ATTCAAGCAGCTATGGGATTCAA	CTGGACGAAGATCGCGTCTG
SKIL	GAGGCTGAATATGCAGGACAG	CTATCGGCCTCAGCATGG
TNFα	AACCTCCTCTCTGCCATCAA	ATGTTCGTCCTCCTCACAGG
TNFSF10	TGGCAACTCCGTCAGCTCGTTA	AGCTGCTACTCTCTGAGGACCT

Table S3. siRNA sequences.

Gene	Sense	Antisense
si-Ctrl	UUCUCCGAACGUGUCACGUTT	ACGUGACACGUUCGGAGAATT
si-CDK1 #1	GGCACUGAAUCAUCCAUAUTT	AUAUGGAUGAUUCAGUGCCTT
si-CDK1 #2	GCACUCCCAAUAAUGAAGUTT	ACUUCAUUAUUGGGAGUGCTT
si-CDK1 #3	GUCUUCAGGAUGUGCUUAUTT	AUAAGCACAUCCUGAAGACTT

Table S4. GSEA (AGING gene set).

AREG	CXCR6	IL31	SERPINB1	BCL10	IFI27L1	PLCH2	TERF1	SLC6A2
AXL	FGF16	IL31RA	SERPINB10	BCL11A	IFI27L2	PML	TERF2	SLC6A9
BMP1	FGF17	IL32	SERPINB11	BCL11B	IFI30	POLG	TFAP2A	SLC7A8
BMP10	FGF18	IL33	SERPINB12	BCL2	IFI35	PPARGC1A	TGFB1	SLC9A3
BMP15	FGF19	IL34	SERPINB13	BCL2L14	IFI44	PPARGC1B	TGFB1I1	SLC9A9
BMP2	FGF21	IL36A	SERPINB2	BCL2L2	IFI44L	PRKAA2	TGFB2	SLCO3A1
BMP3	FGF7	IL36B	SERPINB3	BCL3	IFI6	PRKAB1	TGFB2-AS1	
BMP4	FGF8	IL36G	SERPINB4	BMI1	IFIH1	PRKAB2	TGFB2-OT1	
BMP5	GDF1	IL36RN	SERPINB5	BSCL2	IFIT1	PRKACA	TGFB3	
BMP6	GDF10	IL37	SERPINB6	CDKN1A	IFIT1B	PRKACB	TGFBR2	
BMP7	GDF11	IL3RA	SERPINB7	CDKN2A	IFIT2	PRKAG1	TIMM23	
BMP7-AS1	GDF15	IL4	SERPINB8	CDKN2B	IFIT3	PSEN1	TIMM23B	
BMPER	GDF2	IL4I1	SERPINB9	CEBPA	IFIT5	PSEN2	TIMM9	
CCL1	GDF3	IL4R	SERPINB9P1	CEBPB	IFITM1	PTEN	TIMMDC1	
CCL11	GDF5	IL5	SERPINC1	CEBPD	IFITM10	PTGS1	TIMP1	
CCL13	GDF6	IL5RA	SERPIND1	CETP	IFITM2	PTGS2	AREG	
CCL14	GDF7	IL6	SERPINE1	CHCHD1	IFITM3	PYCR1	AXL	
CCL15	GDF9	IL6R	SERPINE2	CHCHD10	IFITM4P	PYCR2	TOP1	
CCL15-CCL14	HGF	IL6ST	SERPINE3	CHEK2	IFITM5	RECQL	TP53	
CCL16	IGF1	IL7R	SERPINF1	CREB1	IGF1	RECQL4	TP53I11	
CCL17	IGF1R	IL9	SERPINF2	CREB3	IGF1R	RECQL5	TP53I3	
CCL18	IGF2	ITGA1	SERPING1	CREBBP	IGF2	REL	TP53INP1	
CCL19	IGF2-AS	ITGA10	SERPINH1	CSF2RB	IGF2BP1	RELA	TP53	
CCL2	IGF2BP1	ITGA11	SERPINI1	CSNK1E	IGF2BP2	RELB	TP53I11	
CCL20	IGF2BP2	ITGA3	SERPINI2	CSNK1G1	IGF2R	RELL1	TP53I3	
CCL21	IGF2R	ITGA4	TNFRSF10A	CTF1	IRS1	RELL2	TP53INP1	
CCL22	IGFALS	ITGA5	TNFRSF10B	CTNNB1	ITGA1	RIPK1	TP63	
CCL23	IGFBP1	ITGA6	TNFRSF10C	CTNND1	ITGA10	RIPK2	TRAF3	
CCL24	IGFBP2	ITGA8	TNFRSF10D	DBN1	ITGA11	RIPK3	TRAF3IP2	
CCL25	IGFBP3	ITGA9	TNFRSF11A	DCTN1	ITGA2	RPS6KA1	TRAF6	
CCL26	IGFBP4	ITGA9-AS1	TNFRSF11B	DDIT3	ITGA2B	RPS6KA2	TRAP1	
CCL27	IGFBP5	ITGAD	TNFRSF12A	DDIT4	ITGA3	RPS6KA2-AS1	TRIM14	
CCL28	IGFBP6	ITGAE	TNFRSF13B	DDX58	ITGA4	RPS6KA2-IT1	TRIM16	
CCL3	IGFBP7	ITGAL	TNFRSF13C	DDX60L	JUN	RPS6KA5	TRIM16L	
CCL3L1	IL10	ITGAM	TNFRSF14	EDARADD	JUND	S100A1	TRIM21	
CCL3L3	IL10RA	ITGAV	TNFRSF17	EGF	JUP	S100A10	TRIM22	
CCL4	IL10RB	ITGAX	TNFRSF18	EGR1	KLF3	S100A11	TRIM25	
CCL4L1	IL10RB-AS1	ITGB1	TNFRSF19	EGR2	KLF4	S100A12	TRIM36	
CCL4L2	IL11	ITGB1BP1	TNFRSF1A	EGR3	KLF5	S100A13	TRIM37	
CCL5	IL11RA	ITGB1BP2	TNFRSF1B	EGR4	KLF6	S100A14	TRIM38	
CCL7	IL12A	ITGB2	TNFRSF21	EIF4EBP1	KLF7	S100A16	TRIM4	
CCL8	IL12A-AS1	ITGB2-AS1	TNFRSF4	EPOR	KRAS	S100A2	TRIM46	
CD47	IL12B	ITGB6	TNFRSF6B	ERCC1	LMNB1	S100A3	TRIM47	
CD55	IL12RB1	ITGB8	TNFRSF8	ESR1	LRP1	S100A7	TRIM5	
CD58	IL12RB2	MMP1	TNFRSF9	FADS1	LRP10	S100A7A	TRIM6	
CD59	IL13	MMP10	TNFSF10	FADS3	LRP11	S100P	TRPV1	
CD6	IL13RA1	MMP11	TNFSF11	FAS	MAP3K4	SDHC	SLC10A7	
CD63	IL13RA2	MMP12	TNFSF12	FAS-AS1	MAP3K5	SESN1	SLC11A2	
CD68	IL15	MMP13	TNFSF12-TNFSF13	FGF1	MAP3K8	SESN2	SLC12A2	
CD69	IL16	MMP14	TNFSF13	FGF11	MAP3K9	SESN3	SLC12A4	
CD74	IL17A	MMP19	TNFSF13B	FGF13	MAX	SHC1	SLC12A6	
CD81	IL17B	MMP2	TNFSF14	FGF2	MDM2	SIN3A	SLC12A8	
CD83	IL17C	MMP20	TNFSF15	FGF5	MSRA	SIRT1	SLC15A3	
CD9	IL17RA	MMP21	TNFSF18	FGFBP1	MSRB1	SIRT2	SLC16A1	
CD93	IL1A	MMP23A	TNFSF4	FGFR2	MSRB2	SIRT3	SLC16A13	
CD96	IL1B	MMP23B	TNFSF8	FOS	MXD1	SIRT4	SLC16A8	
CD99	IL1F10	MMP26	TNFSF9	FOSB	MXD3	SIRT5	SLC16A9	
CSF1	IL1R1	MMP27	ADH5	FOSL1	MXD4	SIRT6	SLC1A4	
CSF1R	IL1R2	MMP28	ADH7	FOSL2	MXRA5	SIRT7	SLC20A2	
CSF2	IL1RAP	MMP3	AGPAT4	FOXL1	MXRA7	SNCG	SLC22A13	
CSF2RA	IL1RAPL1	MMP7	APOC3	FOXL2	MYC	SOCS1	SLC22A20	
CSF2RB	IL1RAPL2	MMP8	APOE	FOXL2NB	NBN	SOCS2	SLC24A1	
CSF3	IL1RL1	OAS1	APOF	GCK	NFE2	SOCS2-AS1	SLC25A12	
CSF3R	IL1RL2	OAS2	APOH	GPX1	NFE2L1	SOCS3	SLC25A21	
CXCL1	IL1RN	OAS3	APOL1	GPX2	NFKB1	SOCS4	SLC25A21-AS1	
CXCL10	IL2	OASL	APOL2	GPX3	NFKBIA	SOCS5	SLC25A25-AS1	
CXCL11	IL21	PTGES	APOL3	GPX8	NGFR	SOCS6	SLC25A28	
CXCL12	IL21-AS1	RPS6KA2	APOL4	GRN	NGFRAP1	SOCS7	SLC25A29	
CXCL13	IL21R	RPS6KA3	APOL5	GSK3B	NLRP2	SOD1	SLC26A1	
CXCL14	IL21R-AS1	RPS6KC1	APOL6	GSS	NR3C1	SOD2	SLC26A11	
CXCL16	IL22	SERP1	AREG	GSTA4	NR4A1	SOD3	SLC28A3	
CXCL17	IL22RA1	SERP2	ARNTL	HESX	NR4A3	OAS1	SLC2A6	
CXCL2	IL22RA2	SERPINA1	ARNTL2	HELLS	NRG1	OAS2	SLC2A9	
CXCL3	IL23A	SERPINA10	ARNTL2-AS1	HGF	PAPPA	OAS3	SLC30A9	
CXCL5	IL23R	SERPINA11	ATF5	HIF1A	PIAS1	OASL	SLC36A1	
CXCL6	IL24	SERPINA12	ATG10	HSP90AA1	PIAS2	STAT1	SLC36A4	
CXCL8	IL25	SERPINA13P	ATG101	HSP90B2P	PIAS3	STAT2	SLC38A6	
CXCL9	IL26	SERPINA2	ATG14	HSPA12A	PIAS4	STAT3	SLC39A11	
CXCR1	IL27	SERPINA3	ATG2A	HSPA14	PIK3C2G	STAT4	SLC39A8	
CXCR2	IL27RA	SERPINA4	ATG2B	HSPA1A	PIK3CA	STAT5A	SLC39A9	
CXCR2P1	IL2RA	SERPINA5	ATG4A	HSPA4L	PIK3CD	STAT5B	SLC5A5	
CXCR3	IL2RB	SERPINA6	ATM	HTT	PLAU	STAT6	SLC6A14	
CXCR4	IL2RG	SERPINA7	BAK1	IFI16	PLAUR	STUB1	SLC6A15	
CXCR5	IL3	SERPINA9	BAX	IFI27	PLCG2	TAB2	SLC6A16	

Table S5. GSEA (SASP gene set).

AREG	CCL7	CXCR2P1	IL10RB	IL25	ITGAE	SERPINA1	TNFRSF10D
AXL	CCL8	CXCR3	IL10RB-AS1	IL26	ITGAL	SERPINA10	TNFRSF11A
BMP1	CD47	CXCR4	IL11	IL27	ITGAM	SERPINA11	TNFRSF11B
BMP10	CD55	CXCR5	IL11RA	IL27RA	ITGAV	SERPINA12	TNFRSF12A
BMP15	CD58	CXCR6	IL12A	IL2RA	ITGAX	SERPINA13P	TNFRSF13B
BMP2	CD59	FGF16	IL12A-AS1	IL2RB	ITGB1	SERPINA2	TNFRSF13C
BMP3	CD6	FGF17	IL12B	IL2RG	ITGB1BP1	SERPINA3	TNFRSF14
BMP4	CD63	FGF18	IL12RB1	IL3	ITGB1BP2	SERPINA4	TNFRSF17
BMP5	CD68	FGF19	IL12RB2	IL31	ITGB2	SERPINA5	TNFRSF18
BMP6	CD69	FGF21	IL13	IL31RA	ITGB2-AS1	SERPINA6	TNFRSF19
BMP7	CD74	FGF7	IL13RA1	IL32	ITGB6	SERPINA7	TNFRSF1A
BMP7-AS1	CD81	FGF8	IL13RA2	IL33	ITGB8	SERPINA9	TNFRSF1B
BMPER	CD83	GDF1	IL15	IL34	MMP1	SERPINB1	TNFRSF21
CCL1	CD9	GDF10	IL16	IL36A	MMP10	SERPINB10	TNFRSF4
CCL11	CD93	GDF11	IL17A	IL36B	MMP11	SERPINB11	TNFRSF6B
CCL13	CD96	GDF15	IL17B	IL36G	MMP12	SERPINB12	TNFRSF8
CCL14	CD99	GDF2	IL17C	IL36RN	MMP13	SERPINB13	TNFRSF9
CCL15	CSF1	GDF3	IL17RA	IL37	MMP14	SERPINB2	TNFSF10
CCL15-CCL14	CSF1R	GDF5	IL1A	IL3RA	MMP19	SERPINB3	TNFSF11
CCL16	CSF2	GDF6	IL1B	IL4	MMP2	SERPINB4	TNFSF12
CCL17	CSF2RA	GDF7	IL1F10	IL4I1	MMP20	SERPINB5	TNFSF12-TNFSF13
CCL18	CSF2RB	GDF9	IL1R1	IL4R	MMP21	SERPINB6	TNFSF13
CCL19	CSF3	HGF	IL1R2	IL5	MMP23A	SERPINB7	TNFSF13B
CCL2	CSF3R	IGF1	IL1RAP	IL5RA	MMP23B	SERPINB8	TNFSF14
CCL20	CXCL1	IGF1R	IL1RAPL1	IL6	MMP26	SERPINB9	TNFSF15
CCL21	CXCL10	IGF2	IL1RAPL2	IL6R	MMP27	SERPINB9P1	TNFSF18
CCL22	CXCL11	IGF2-AS	IL1RL1	IL6ST	MMP28	SERPINC1	TNFSF4
CCL23	CXCL12	IGF2BP1	IL1RL2	IL7R	MMP3	SERPIND1	TNFSF8
CCL24	CXCL13	IGF2BP2	IL1RN	IL9	MMP7	SERPINE1	TNFSF9
CCL25	CXCL14	IGF2R	IL2	ITGA1	MMP8	SERPINE2	TGFB1
CCL26	CXCL16	IGFALS	IL21	ITGA10	OAS1	SERPINE3	TGFB2
CCL27	CXCL17	IGFBP1	IL21-AS1	ITGA11	OAS2	SERPINF1	TGFB3
CCL28	CXCL2	IGFBP2	IL21R	ITGA3	OAS3	SERPINF2	
CCL3	CXCL3	IGFBP3	IL21R-AS1	ITGA4	OASL	SERPING1	
CCL3L1	CXCL5	IGFBP4	IL22	ITGA5	PTGES	SERPINH1	
CCL3L3	CXCL6	IGFBP5	IL22RA1	ITGA6	RPS6KA2	SERPINI1	
CCL4	CXCL8	IGFBP6	IL22RA2	ITGA8	RPS6KA3	SERPINI2	
CCL4L1	CXCL9	IGFBP7	IL23A	ITGA9	RPS6KC1	TNFRSF10A	
CCL4L2	CXCR1	IL10	IL23R	ITGA9-AS1	SERP1	TNFRSF10B	
CCL5	CXCR2	IL10RA	IL24	ITGAD	SERP2	TNFRSF10C	
